# Strain differences in thymic atrophy in rats immunized for EAE correlate with the clinical outcome of immunization

**DOI:** 10.1371/journal.pone.0201848

**Published:** 2018-08-07

**Authors:** Mirjana Nacka-Aleksić, Marija Stojanović, Ivan Pilipović, Zorica Stojić-Vukanić, Duško Kosec, Gordana Leposavić

**Affiliations:** 1 Department of Physiology, University of Belgrade - Faculty of Pharmacy, Belgrade, Serbia; 2 Immunology Research Centre “Branislav Janković”, Institute of Virology, Vaccines and Sera “Torlak”, Belgrade, Serbia; 3 Department of Microbiology and Immunology, University of Belgrade - Faculty of Pharmacy, Belgrade, Serbia; Queen Mary University of London, UNITED KINGDOM

## Abstract

An accumulating body of evidence suggests that development of autoimmune pathologies leads to thymic dysfunction and changes in peripheral T-cell compartment, which, in turn, perpetuate their pathogenesis. To test this hypothesis, thymocyte differentiation/maturation in rats susceptible (Dark Agouti, DA) and relatively resistant (Albino Oxford, AO) to experimental autoimmune encephalomyelitis (EAE) induction was examined. Irrespective of strain, immunization for EAE (i) increased the circulating levels of IL-6, a cytokine causally linked with thymic atrophy, and (ii) led to thymic atrophy reflecting partly enhanced thymocyte apoptosis associated with downregulated thymic IL-7 expression. Additionally, immunization diminished the expression of Thy-1, a negative regulator of TCRαβ-mediated signaling and activation thresholds, on CD4+CD8+ TCRαβ^lo/hi^ thymocytes undergoing selection and thereby impaired thymocyte selection/survival. This diminished the generation of mature CD4+ and CD8+ single positive TCRαβ^hi^ thymocytes and, consequently, CD4+ and CD8+ recent thymic emigrants. In immunized rats, thymic differentiation of natural regulatory CD4+Foxp3+CD25+ T cells (nTregs) was particularly affected reflecting a diminished expression of IL-7, IL-2 and IL-15. The decline in the overall thymic T-cell output and nTreg generation was more pronounced in DA than AO rats. Additionally, differently from immunized AO rats, in DA ones the frequency of CD28- cells secreting cytolytic enzymes within peripheral blood CD4+ T lymphocytes increased, as a consequence of thymic atrophy-related replicative stress (mirrored in CD4+ cell memory pool expansion and p16^INK4a^ accumulation). The higher circulating level of TNF-α in DA compared with AO rats could also contribute to this difference. Consistently, higher frequency of cytolytic CD4+ granzyme B+ cells (associated with greater tissue damage) was found in spinal cord of immunized DA rats compared with their AO counterparts. In conclusion, the study indicated that strain differences in immunization-induced changes in thymopoiesis and peripheral CD4+CD28- T-cell generation could contribute to rat strain-specific clinical outcomes of immunization for EAE.

## Introduction

The thymus is the primary organ providing microenvironment for T-cell development. The continual output of mature T cells to the periphery maintains the diversity of the peripheral T-cell repertoire [1]. The thymus also regulates T-cell homeostasis by clonal deletion of self-reactive effector T cells during negative selection [2,3]. This process is not immaculate and some autoreactive cells inevitably escape to the periphery. However, the thymus is also a source of natural regulatory CD4+ T cells (nTregs) that control autoreactive T cells escaping negative selection [4–6].

Considering the aforementioned, it is clear that the diminished thymopoiesis occurring during aging, mirrored in the reduced frequency of recent thymic emigrants (RTEs), leads to contraction of the peripheral naïve T-cell repertoire. However, due to homeostatic expansion the overall peripheral T-cell count remains stable [7,8]. Homeostatic proliferation of autoreactive T cells has been associated with higher propensity for development of autoimmunity in the elderly [9,10]. However, this does not translate into an increased incidence of autoimmune diseases in aging [11]. This phenomenon was associated with an aging-related expansion of protective T-cell regulatory mechanisms in the periphery [11,12] and age-related intrinsic T-cell changes underlying their functional erosion [13].

On the other hand, a premature thymic involution has been linked with the development of human inflammatory autoimmune diseases, including multiple sclerosis (MS) [14–16]. To explain this phenomenon, an increased T-cell homeostatic proliferation due to premature decline in the overall thymic T-cell output, in conjunction with Treg dysregulation due to a prominent decrease in nTreg output, has been proposed [17,18]. Additionally, an increase in the frequency of CD28- T cells, which are resistant to immunoregulation and exhibit cytolytic and proinflammatory properties, is suggested to contribute to development of autoimmune diseases in premature immune aging [19,20].

It should also be pointed that development of inflammatory autoimmune diseases leads to a premature decline in thymopoietic efficiency [21,22] and loss of CD28 expression on CD4+ T cells, reflecting not only a reduced thymopoiesis, but also a direct inhibitory action of proinflammatory cytokines on its expression [23,24]. These pathogenetic events, in turn, contribute to perpetuation of these diseases [21–24]. Thus, the rise in the frequency of CD4+CD28- T cells has been proposed as a biomarker of not only chronobiological, but also of „premature aging”of the immune system under such pathological conditions [23,24]. Moreover, it was shown that the frequency of CD4+CD28- cells (exhibiting persistent activation and memory T cell phenotype) in peripheral blood correlates with the clinicopathological features of inflammatory autoimmune diseases [25]. It should be noted that most studies on CD4+CD28- T cells have been conducted in humans and that development of appropriate animal models is required in order to investigate the underlying mechanisms of this phenomenon and its biological relevance *in vivo* [23].

Additionally, it has been shown that the development of various autoimmune pathologies in experimental animals, including experimental autoimmune encephalomyelitis (EAE), the most common experimental model for MS [26], also leads to thymic atrophy and decline in thymopoiesis [27,28]. Multiple cellular and molecular pathways are supposed to be involved in thymic atrophy in experimental autoimmune diseases, of which some could be common to those contributing to age-related thymic involution [29]. Moreover, an accumulating body of evidence indicates that the thymic changes, in turn, contribute to pathogenesis of autoimmune diseases [30]. Consistently, it has been assumed that immunological abnormalities in not only peripheral lymphoid tissues and the target organs, but also in the thymus participate in autoimmune disease development [31].

To understand the thymic changes in autoimmune diseases, a brief overview of T-cell differentiation/maturation is necessary. Within the thymus, blood-borne CD4-CD8- double negative (DN) thymocyte precursors undergo rearrangement of the T-cell receptor (TCR) β gene, a process known as β selection [32]. The product of the properly rearranged TCRβ gene along with CD3 and the pre-Tα protein forms the pre-TCR complex [32]. This leads to upregulation of CD4 and CD8 expression and development of CD4+CD8+ double positive (DP) cells [33,34], TCRα locus rearrangement and formation of functional TCRαβ complex. These DP TCRαβ^lo^ thymocytes are scrutinized for their ability to recognize peptides in the context of self-MHC expressed on thymic stromal cells [33,34]. The cells transducing moderate TCRαβ signal strength (positive selection) continue to differentiate into mature CD4+CD8- or CD4-CD8+ single positive (SP) TCRαβ^hi^ cells that emigrate from the thymus, forming a pool of RTEs in the periphery. All other cells terminate differentiation being eliminated by either negative selection (those transducing a strong TCRαβ signal) or dying by neglect (those transducing weak TCRαβ signal). Thus, the relationship between the relative proportions of distinct thymocyte subsets expressing characteristic constellation of phenotypic markers provides insight into the complex process of T-cell differentiation/maturation.

It should be pointed out that aging leads not only to quantitative, but also qualitative changes in thymopoiesis mirrored in alterations in the thymocyte subset distribution [35]. It increases the proportion of DN cells, but diminishes that of DP cells [36,37]. Also, in aged rats a disruption in TCR rearrangement and β selection, affecting the transition from DN to DP thymocyte developmental stage, along with disrupted positive/negative selection of thymocytes has been observed [37,38].

It is noteworthy that although strain differences in the size of the thymus and its epithelial compartment have been observed [39–41], strain differences in thymopoiesis have not been investigated, yet. More important in the context of autoimmune disease development, there are no data on the influence of the genetic background of experimental animals on thymic changes associated with development of autoimmune pathology.

Having all the aforementioned in mind, female Dark Agouti (DA) rats (susceptible to EAE induction) and Albino Oxford (AO) rats (relatively resistant to EAE induction) [42] were examined for the influence of immunization for EAE on: i) quantitative and qualitative characteristics of thymopoiesis (generation of distinct subpopulation of conventional T cells and CD4+ nTregs), and ii) the phenotypic profile of the main subpopulations of T-peripheral blood lymphocytes (T-PBLs) in terms of the frequency of RTEs, as an indicator of thymopoietic efficacy [43], and CD28- T cells, as their accumulation, which could also be associated with thymic atrophy, contributes to target tissue damage [44]. Additionally, to elucidate the putative mechanisms underlying thymopoietic changes, thymic tissue was examined for the expression of molecules regulating thymocyte precursor cell entry into the thymus (CXCL12) [45], their survival and differentiation/maturation (IL-7, IL-6) [46–49], and differentiation/maturation of CD4+ nTregs (IL-2, IL-15) [50]. Moreover, the circulating level of the proinflammatory cytokines IL-6 (associated with the decline in the efficacy of thymopoiesis [49]) and TNF-α, which is shown to induce not only thymic atrophy [51], but also loss of CD28 expression on CD4+ T cells [23,24,52] was investigated.

## Materials and methods

### Experimental animals

Young adult (2-3-month-old) female DA and AO rats from a breeding colony of the Immunology Research Centre “Branislav Janković”in Belgrade were used in the present study. The animal facilities were endorsed by the Ministry of Agriculture and Enviromental Protection of the Republic of Serbia (Veterinary Department). Animals were bred in standard laboratory conditions (3 rats/polyethylene cage containing sterilized wood shavings, under controlled humidity, temperature and lighting conditions) and provided with standard food and fresh water *ad libitum*. This study was carried out in strict accordance with the the directive 2010/63/EU of the European Parliament and the Council on the protection of animals used for scientific purposes (revising Directive 86/609/EEC) and the governmental regulations (Law on Animal Welfare, “Official Gazette of RS”, no. 41/2009). The study protocol was approved by the Animal Care and Use Committee of the Faculty of Pharmacy (Etički komitet Farmaceutskog fakulteta, permit number 6/12). Animal health monitoring was performed on a daily basis by animal care staff and a veterinarian. All procedures aforementioned are in accordance to the ARRIVE Guidelines for reporting animal research and the completed ARRIVE guidelines checklist is included as S1 File.

### Induction and clinical evaluation of EAE

Rats of both strains were randomly assigned to immunization (“immunized for EAE” group) or left untreated (“non-immunized” group). For induction of EAE, rats received 100 μl of an emulsion made of equal volumes of rat spinal cord (SC) homogenate in phosphate-buffered saline (PBS) and complete Freund’s adjuvant (CFA) containing 1 mg/ml of heat-killed and dried *Mycobacterium tuberculosis* H37Ra (Sigma-Aldrich Chemie GmbH, Taufkirchen, Germany) into the left hind foot pad, and 0.25 ml of saline suspension of 5 × 10^8^ *Bordetella pertussis* (Institute of Virology, Vaccines and Sera “Torlak“, Belgrade, Serbia) subcutaneously into the dorsum of the same paw [42]. To diminish stress, pain and injury, rats were administered an intraperitoneal injection of ketamine (Ketamidor, Richter Pharma AG, Wels, Austria; 100 mg/ml)/xylazine (Xylased, Bioveta, Ivanovice na Hané, Czech Republic; 20 mg/ml) anesthetizing cocktail [50 mg/kg body weight (BW) of ketamine/5 mg/kg BW xylazine]. The weight of the animals and their EAE score (0, no clinical signs; 0.5, distal tail atony; 1, complete tail atony; 2, paraparesis; 3, paraplegia; 4, tetraplegia or moribund state) were recorded daily by two independent experienced observers, starting at the 1^st^ day post-immunization (d.p.i.). None of the diseased rats reached moribundity during the studies. To improve welfare and clinical status, the animals which developed clinically manifested EAE had their mashed food and water positioned lower to facilitate access. When DA rats reached the peak of the disease on the 13^th^ d.p.i. [42,53], all animals were sacrificed through transcardial perfusion. Before the perfusion the animals were deeply anesthetized with an intraperitoneal injection of ketamine/xylazine anesthetizing cocktail (80 mg/kg BW ketamine/8 mg/kg BW xylazine) and blood samples were taken by cardiac puncture. Two sets of experiments were performed. In the first set of experiments the influence of immunization on thymus weight and thymocyte number, thymosuppresive/thymostimulatory cytokines, CXCL12 expression and thymopoiesis, number of T lymphocytes and their expression of CD28, infiltration of spinal cord with CD4+ granzyme B+ T lymphocytes, and circulating levels of inflammatory cytokines was examined (6 rats/group/strain; total 24 rats/strain). In the second set of experiments the putaive influence of immunization-induced thymic atrophy on CD28 loss on T cells (frequency of activated and proliferating cells among the major T lymphocyte subsets, p16^INK4a^ expression in CD4+ and CD8+ T-PBLs) was examined (6 rats/group/strain; total 24 rats/strain). The incidence and clinical course of the disease were similar in both sets of experiments.

### Antibodies and immunoconjugates

The following monoclonal antibodies (mAbs), secondary reagents and isotype controls used in the study were purchesed from BD Biosciences Pharmingen (Mountain View, CA, USA): phycoerythrin (PE)-conjugated anti-CD4 (clone OX-38), peridinin–chlorophyll–protein (PerCP)-conjugated anti-CD90 (Thy-1.1, clone OX-7), fluorescein isothiocyanate (FITC)/PE/biotin/allophycocyanin (APC)-conjugated anti-CD8 (clone OX-8), PerCP-conjugated anti-TCRαβ (clone R73), PE-conjugated anti-CD45RC (clone OX-22), FITC-conjugated anti-CD2 (clone OX-34), FITC/Alexa Fluor^®^ 488-conjugated anti-Ki67 (clone B56), PE-conjugated anti-CD28 (clone JJ319), PerCP-conjugated streptavidin and FITC-conjugated goat anti-mouse Ig Ab. Additionaly, FITC-conjugated anti-Foxp3 (clone FJK-16s), PerCP eFluor^®^ 710-conjugated anti-CD25 (clone OX-39), APC-conjugated anti-CD4 (clone OX-35) (all eBioscience, San Diego, CA, USA), FITC-conjugated anti-granzyme B (clone GB11), and Alexa Fluor^®^ 647-conjugated anti-TCRαβ (clone R73) (both BioLegend, San Diego, CA, USA) mAbs were also used. Unconjugated polyclonal anti-CD69 (P-17) Ab was obtained from Santa Cruz Biotechnology (Santa Cruz, CA, USA), FITC-conjugated rabbit anti-goat IgG Ab and merocyanine dye (MC540) from Sigma–Aldrich Chemie GmbH, and unconjugated mouse anti-CDKN2A/p16INK4a (clone 2D9A12) mAb from Abcam (Cambridge, UK).

### Preparation of cell suspensions and plasma samples

To obtain single cell suspensions, fresh thymus or SC tissue was finely minced and pressed through 70 μm nylon cell strainers (BD Biosciences, Erembodegem, Belgium). The obtained thymic cell suspensions were washed three times in ice-cold PBS supplemented with 2% fetal calf serum (FCS, Gibco, Grand Island, NY, USA) and 0.1% sodium azide (FACS buffer). The cells from the SCs were collected in RPMI 1640 medium (Sigma-Aldrich Chemie GmbH) supplemented with 5% FCS and fractioned on a discontinuous 40/70% Percoll (Sigma-Aldrich Chemie GmbH) gradient at 1000 × *g* for 50 min. Mononuclear cells from the interface were collected. For quantification of T-PBLs, erythrocyte lysis was performed by adding isotonic solution of ammonium chloride to blood samples in 1:5 volume ratio. The tubes were occasionally shaken mildly and then centrifuged at 350 × *g* for 5 min at 4°C. Thus obtained cells were washed three times in ice cold PBS supplemented with 2% FSC.

All cells were enumerated using improved Neubauer hemocytometer.

The blood samples for plasma preparation were stored at 4°C for 30 minutes and then centrifuged for 15 min at 1500 × *g*. Plasma was collected in mini tubes and stored at -70°C until analysis.

### Detection of apoptotic thymocytes

One hundred μl of freshly isolated (5 × 10^6^ cells/ml) thymocytes was plated in 96-well flat-bottom plates (Nunc A/S, Roskilde, Denmark) in culture medium [RPMI 1640 medium supplemented with 2 mM L-glutamine (Serva, Heidelberg, Germany), 1 mM sodium pyruvate (Serva), 100 units/ml penicillin (ICN, Costa Mesa, CA, USA), 100 μg/ml streptomycin (ICN) and 10% FCS]. Cells were incubated for 18 h, harvested and 5 μl of MC 540 stock per sample was added to the thymocyte suspension. All samples were then subjected to flow cytometric analysis (FCA).

### RT-qPCR

Thymic tissue samples were collected using Nucleic Acid Purification Lysis Solution (Applied Biosystems, Foster City, CA, USA) and immediately stored at -70°C until RNA purification. Total RNA from tissue samples was extracted using ABI Prism 6100 Nucleic Acid Prep Station system (Applied Biosystems) and Total RNA Chemistry Starter Kit (Applied Biosystems), including DNAse (Absolute RNA Wash Solution, Applied Biosystems) treatment to ensure that no genomic DNA contamination was present. High Capacity cDNA Reverse Transcription Kit (Applied Biosystems) was used for cDNA synthesys, followed by triplicate RT-qPCR reactions performed using TaqMan Gene Expression Master Mix (Applied Biosystems) and predesigned TaqMan Gene Expression assays (Applied Biosystems) in 7500 Real-Time PCR System under pre-optimized conditions. The following TaqMan Gene Expression Assays were used in the study: Il7 (Rn00681900-m1), Il2 (Rn00587673_m1), Il15 (Rn00689963_g1), Il6 (Rn99999011_m1) and Cxcl12 (Rn00573260_m1). Target mRNA levels were quantified by the comparative threshold cycle (dCt) method using SDS v1.4.0. software (Applied Biosystems) and β-actin (Actb; Rn00667869_m1) as a normalizer, as it has been previously suggested [42]. Relative amounts of target mRNAs were given as 2^–dCt^ values, representing the ratio of target to reference gene, where dCt = Ct target − Ct reference.

### Cell staining and FCA

For analyses, 50.000 events per sample were acquired on FACSCalibur or FACSVerse flow cytometer (Becton Dickinson, Mountain View, CA, USA). Data were analyzed for frequency of marker positive cells and/or mean fluorescence intensity (MFI), which represents the density of marker expression, using FlowJo software version 7.8. (TreeStarInc, Ashland, OR, USA). IgG isotype-matched controls were used for each fluorochrome type and fluorescence minus one controls were applied to settle gating boundaries. The MFI values are expressed as MFI ratio [MFI of mAb-labeled cells/MFI of unstained cells (negative control), as previously suggested] [54].

### Surface antigen immunostaining

For antigen immunostaining of thymocytes, SC mononuclear cells and T-PBLs, cells were incubated with saturating concentrations of either fluorochrome-labeled or unconjugated/biotin-conjugated mAbs for 30 min and washed in FACS buffer. When unconjugated/biotin-conjugated mAb was applied the cells were incubated with appropriate second step reagent for additional 30 min, washed and collected for FCA. All incubation steps were performed at 4°C.

### Intracellular antigen immunostaining

After surface antigen immunolabeling, thymocytes and SC cells were fixed/permeabilized using the reagents from the fixation/permeabilization buffer kit (eBioscience), and stained with anti-Foxp3, anti-Ki-67, anti-granzime B or p16^INK4a^/anti-mouse IgG Abs, as suggested by the manufacturer.

### ELISA

Commercial ELISA kits were used for measuring the concentration of IL-6 and TNF-α in rat plasma (BioLegend). A standard curve was generated for each assay with the limit of detection for IL-6 = 5.3 pg/ml and TNF-α = 2 pg/ml. All steps were performed according to the manufacturer’s instruction.

### Statistical analysis

To assess the influence of immunization and rat strain on the immunological parameters two-way ANOVA followed by the Bonferroni *post hoc* test was used. The influence of rat strain on the frequency of granzyme B+ CD4+ cells in SC of rats immunized for EAE was assessed using Student’s t-test. All analyses were performed using GraphPad Prism 5 software (GraphPadSoftware, Inc., La Jolla, CA, USA). Values of p<0.05 were considered significant. All statistical data are displayed in S2 File.

## Results

### Differential clinical outcome of immunization for EAE in DA and AO rats

As previously shown [42], injection of SC homogenate in CFA induced a monophasic, self-limiting debilitating disease in all immunized DA rats, while none of the AO rats developed clinically manifested neurological deficit (S1A Fig).

### Immunization for EAE diminished thymic weight and thymocyte yield to a greater extent in DA rats than in AO ones

**Fig 1:**
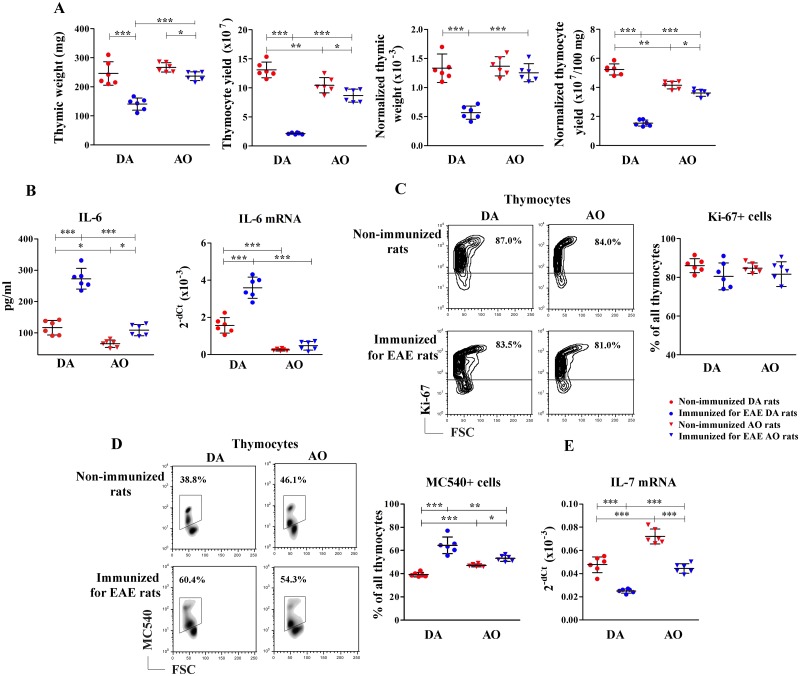
Immunization for EAE reduced the thymic weight and thymocyte yield more effectively in DA than in AO rats. Scatter plots indicate **(A)** thymic weight, thymocyte yield, normalized thymic weight (thymic weight/BW) and normalized thymocyte yield (per 100 mg thymic tissue) and **(B)** circulating levels of IL-6 and thymic expression of IL-6 mRNA in non-immunized and immunized for EAE DA and AO rats. **(C)** Representative flow cytometry contour plots show Ki-67 staining of thymocytes retrieved from non-immunized and immunized for EAE DA and AO rats (gating strategy is displayed in S2A Fig). Scatter plot indicates the frequency of Ki-67+ cells among thymocytes from non-immunized and immunized for EAE DA and AO rats. **(D)** Representative flow cytometry density plots show MC540 staining of thymocytes of non-immunized and immunized for EAE DA and AO rats, cultured for 18h in culture medium to assess cell apoptosis (see Materials and methods). Scatter plot indicates the frequency of MC540+ thymocytes of non-immunized and immunized for EAE DA and AO rats. **(E)** Scatter plot indicates IL-7 mRNA expression in thymic tissue of non-immunized and immunized for EAE DA and AO rats. Two way ANOVA showed significant interaction between the effects of strain and immunization for thymic weight (F_(1,20)_ = 6.954, p<0.01), normalized thymic weight (F_(1,20)_ = 14.63, p<0.01), thymocyte yield (F_(1,20)_ = 69.94, p<0.001), normalized thymocyte yield (F_(1,20)_ = 148.1, p<0.001), frequency of MC540+ thymocytes (F_(1,20)_ = 19.37, p<0.001), circulating levels of IL-6 (F_(1,20)_ = 19.60, p<0.001) and thymic expression of IL-6 mRNA (F_(1,20)_ = 35.57, p<0.001). Data points, means and ± SD are from one of two experiments with similar results (n = 6). * p<0.05; ** p<0.01; *** p<0.001.

In rats of both strains thymopoietic parameters were analyzed on the 13^th^ d.p.i. which corresponds to the peak of EAE in DA rats [42,53]. In immunized DA rats, marked (p<0.001) thymic weight loss was observed compared with strain-matched non-immunized rats (Fig 1A). On the other hand, at the same post-immunization point, in AO rats the thymic weight was only slightly lower (p<0.05) than that in the strain-matched non-immunized ones (Fig 1A). Upon immunization the thymocyte yield dramatically declined (p<0.001) in DA rats, while it was only slightly decreased (p<0.05) in AO ones (Fig 1A). Given that the BW was also reduced in the diseased DA rats (S1B Fig), the thymic weight was normalized to the BW. The normalized thymic weight and the normalized thymocyte yield (per 100 mg thymic weight) were also less (p<0.001) in immunized DA rats than in the strain-matched non-immunized ones (Fig 1A). This suggested thymus-specific influence of immunization in DA rats. In AO animals the normalized thymic weight was similar between the immunized and non-immunized rats, while the normalized thymocyte yield was only slightly (p<0.05) diminished in immunized compared with non-immunized rats (Fig 1A). In non-immunized animals, neither the absolute nor the normalized thymic weight differed between the two rat strains (Fig 1A). However, the absolute and normalized thymocyte yields were less (p<0.01) in non-immunized AO rats than in their DA counterparts (Fig 1A). On the other hand, the absolute and the normalized thymic weights, and the absolute and the normalized thymocyte yields were less (p<0.001) in immunized DA rats than in their AO counterparts (Fig 1A). Thus, the effects of immunization on the absolute and normalized thymic weights, as well as on the absolute and normalized thymocyte yields, were strain-specific (Fig 1A).

In rats of both strains immunization-induced changes in the thymic weight and thymocyte yield were followed by a rise in the circulating levels of IL-6, a thymosupressive/thymolytic cytokine [49], but this effect of immunization was more prominent in DA rats (Fig 1B). Additionally, in immunized DA rats exhibiting more prominent changes in the thymic weight and thymocyte yield, increased (p<0.001) thymic expression of IL-6 mRNA compared with the strain matched non-immunized rats was found (Fig 1B). Irrespective of immunization, the circulating level (p<0.05) of IL-6 and its thymic expression (p<0.001) were lower in AO rats than in DA ones (Fig 1B).

To elucidate the mechanisms underlying the influence of immunization on the thymocyte yield, thymocyte proliferation and apoptosis were examined in rats of both strains. Neither immunization, nor the strain affected the overall frequency of proliferating Ki-67+ cells among thymocytes (Fig 1C). However, immunization markedly increased (p<0.001) the frequency of apoptotic cells among thymocytes from DA rats, while it was less efficient (p<0.05) in this respect in AO ones (Fig 1D). Accordingly, the frequency of apoptotic cells was higher (p<0.001) in non-immunized AO rats than in their DA counterparts (Fig 1D). To the contrary, it was lower (p<0.01) in immunized AO rats than in their DA counterparts (Fig 1D).

Considering that IL-7 is crucial for thymocyte survival and differentiation of immature thymocytes [55], thymic expression of IL-7 mRNA was also examined. Immunization diminished (p<0.001) thymic IL-7 mRNA expression in DA and AO rats (Fig 1E). Irrespective of immunization, its expression was greater (p<0.001) in AO than in DA rats (Fig 1E).

### Influence of immunization for EAE on thymocyte subset composition in DA and AO rats

**Fig 2:**
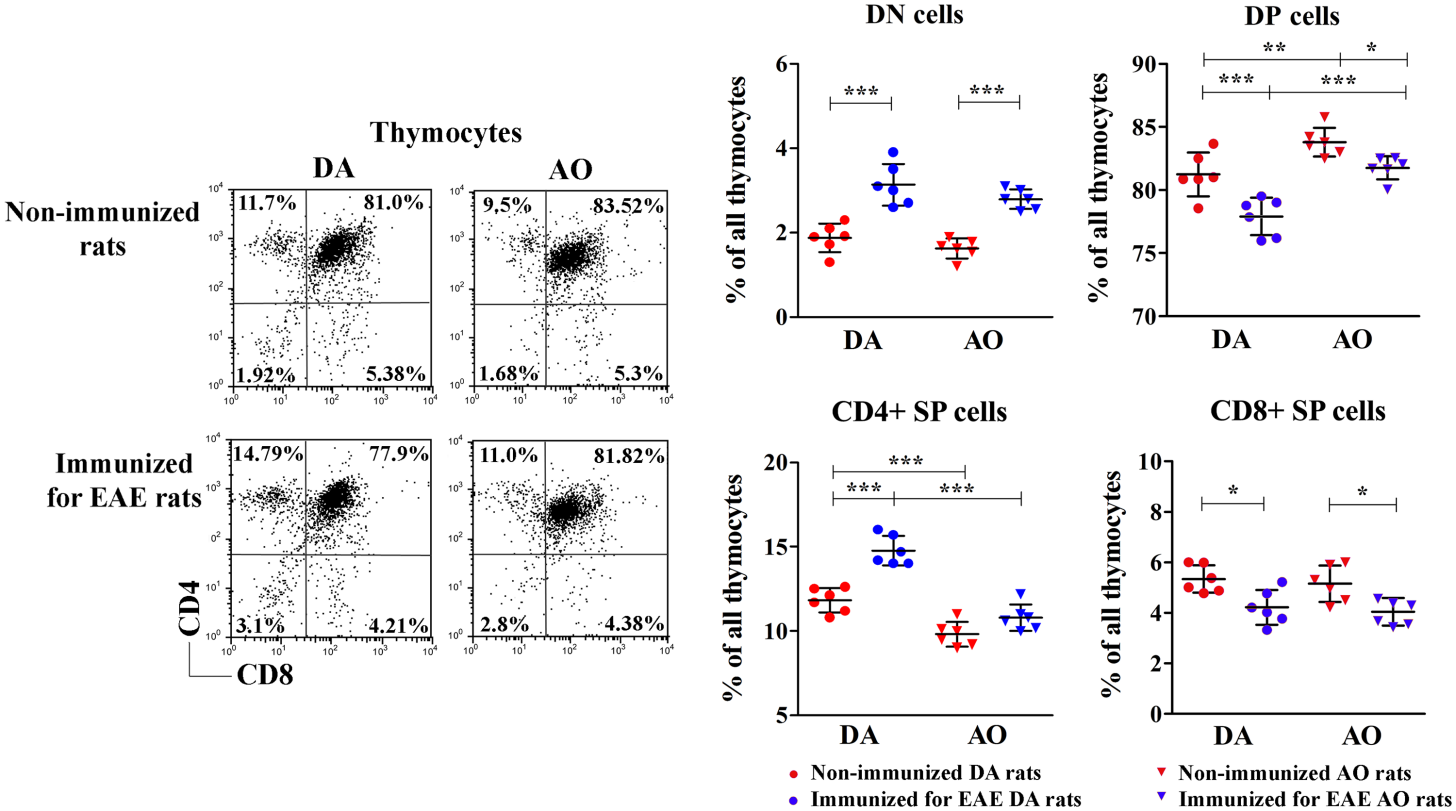
Immunization for EAE affected the composition of thymocyte subsets delineated by CD4/CD8 expression. Representative flow cytometry dot plots show CD4/CD8 staining of thymocytes retrieved from non-immunized and immunized for EAE DA and AO rats (gating strategy is displayed in S3A Fig). Scatter plots indicate the frequency of CD4-CD8- double negative (DN), CD4+CD8+ double positive (DP), and CD4+ and CD8+ single positive (SP) thymocytes from non-immunized and immunized for EAE DA and AO rats. Two way ANOVA showed significant interaction between the effects of strain and immunization for the frequency of CD4+ SP thymocytes (F_(1,20)_ = 9.376, p<0.001). Data points, means and ± SD are from one of two experiments with similar results (n = 6). * p<0.05; ** p<0.01; *** p<0.001.

Over the course of thymocyte “shaping” into mature T cells reactive to foreign antigens and tolerant to self-antigens they pass through distinct differentiational/maturational stages distinguishable by characteristic constellation of surface expression of CD4 and CD8 molecules and the level of TCRαβ molecular complex surface expression [56]. Thus, to evaluate the influence of immunization for EAE on thymocyte differentiation, the frequency of cells in distinct stages of differentiation/maturation was examined. While in non-immunized rats the frequency of DN thymocytes did not differ between DA and AO rats, it increased (p<0.001) in immunized rats of both strains (Fig 2). On the other hand, immunization diminished the frequency of DP thymocytes in DA (p<0.001) and AO (p<0.05) rats, but their frequency remained higher (p<0.001) in immunized AO rats than in their DA counterparts (Fig 2). Immunization increased (p<0.001) the frequency of CD4+ SP thymocytes in DA rats, but it did not significantly affect their frequency in AO ones (Fig 2). Irrespective of immunization, the frequency of SP CD4+ thymocytes was lower (p<0.001) in AO than in DA rats (Fig 2). Differently, immunization diminished (p<0.05) the frequency of CD8+ SP thymocytes in both DA and AO rats, but it remained comparable between these two rat strains (Fig 2).

#### DN thymocyte subset composition

**Fig 3:**
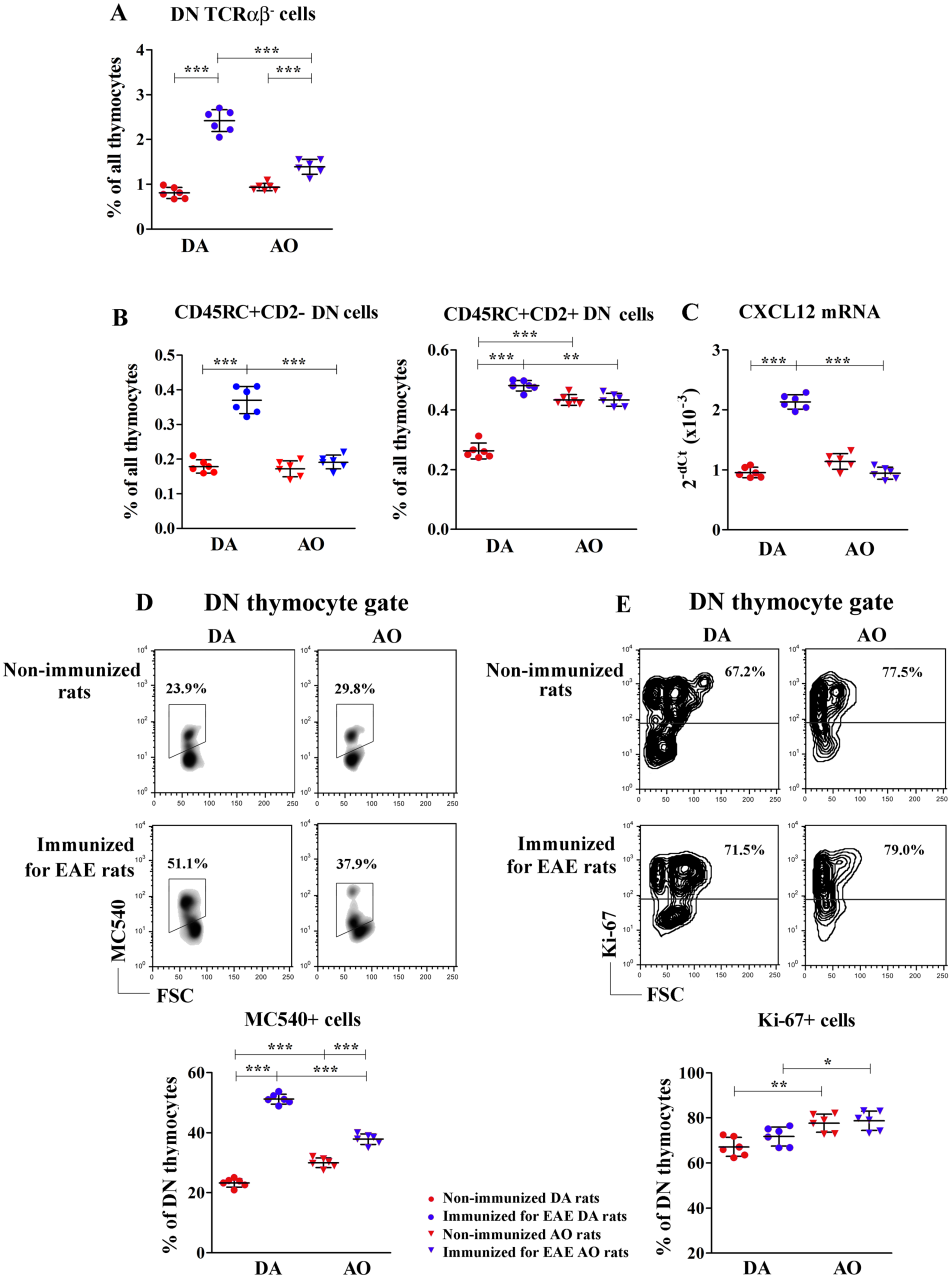
Immunization for EAE affected the frequency of DN TCRαβ^-^ thymocytes in rats of both strains and the frequencies of the least mature CD45RC+CD2- DN and CD45RC+CD2+ DN thymocytes in DA rats. Scatter plots indicate: **(A)** the frequency of CD4−CD8− double negative (DN) TCRαβ^-^ cells among thymocytes from non-immunized and immunized for EAE DA and AO rats (gating strategy is displayed in S3A and S3B Fig); **(B)** the frequency of CD45RC+CD2- and CD45RC+CD2+ DN thymocytes in non-immunized and immunized for EAE DA and AO rats (gating strategy for CD45RC+CD2-/+ cells is displayed in S4 Fig and DN thymocytes are gated as shown in Fig 2) and **(C)** CXCL12 mRNA expression in thymic tissue from non-immunized and immunized for EAE DA and AO rats. **(D)** Representative flow cytometry density plots show MC540 staining of DN thymocytes of non-immunized and immunized for EAE DA and AO rats, cultured for 18h in culture medium to assess cell apoptosis (see Materials and methods). Scatter plot indicates the frequency of apoptotic MC540+ cells within DN thymocytes of non-immunized and immunized for EAE DA and AO rats. **(E)** Representative flow cytometry contour plots show Ki-67 staining of DN thymocytes from non-immunized and immunized for EAE DA and AO rats (gating strategy is displayed in S2B Fig). Scatter plot indicates the frequency of Ki-67+ cells within DN thymocytes of non-immunized and immunized for EAE DA and AO rats. Two way ANOVA showed significant interaction between the effects of strain and immunization for the frequencies of DN TCRαβ^-^ (F_(1,20)_ = 73.35, p<0.001), CD45RC+CD2- DN (F_(1,20)_ = 63.06, p<0.001) and CD45RC+CD2+ DN (F_(1,20)_ = 160.3, p<0.001) cells within thymocytes, the frequency of MC540+ cells within DN thymocytes (F_(1,20)_ = 232.1, p<0.001), and thymic CXCL12 mRNA expression (F_(1,20)_ = 49.48, p<0.001). Data points, means and ± SD are from one of two experiments with similar results (n = 6). * p<0.05; ** p<0.01; *** p<0.001.

Next, to identify the points of dysregulation in the multistep thymopoietic process, we focused on specific subsets of cells within the major four thymocyte subpopulations. Firstly, the frequency of the least mature DN TCRαβ^-^ thymocytes was examined. Immunization markedly increased (p<0.001) the frequency of DN TCRαβ^-^ thymocytes in rats of both strains, but this effect was more prominent in DA rats (Fig 3A). Consequently, although their frequency did not differ between thymocytes from non-immunized DA and AO rats, it was lower (p<0.001) among thymocytes from immunized AO rats compared with their DA counterparts (Fig 3A).

Considering the prominent changes in the proportion of DN TCRαβ^-^ thymocytes following immunization, the frequencies of distinct subsets of DN thymocytes were further analyzed. In the rat, within DN thymocyte subpopulation distinct cell subsets can be delineated according to the surface expression of CD2 and CD45RC molecules [57]. We focused on the frequency of CD45RC+CD2- DN and CD45RC+CD2+ DN cells, which are suggested to have thymopoietic and regenerative properties [57]. Compared with non-immunized strain-matched rats the frequencies of CD45RC+CD2- DN and CD45RC+CD2+ DN cells increased (p<0.001) among thymocytes from immunized DA rats, whereas they remained unaltered in AO rats (Fig 3B). Consequently, the frequency of CD45RC+CD2- DN cells, which did not differ between thymocytes from DA and AO non-immunized rats, was lower (p<0.001) in immunized AO rats when compared with their DA counterparts (Fig 3B). On the other hand, although the frequency of CD45RC+CD2+ DN cells was markedly higher (p<0.001) among thymocytes from non-immunized AO rats compared with DA ones, it was lower (p<0.01) in immunized AO than in immunized DA rats (Fig 3B).

To explain the effects of immunization on the composition of the DN subset, the expression of mRNA for CXCL12, an indispensable factor for proper localization of early lymphoid progenitors in the cortex and consequently their successful steady state differentiation [45], was analyzed. In DA rats immunization upregulated (p<0.001) thymic CXCL12 mRNA expression, while in AO rats it was ineffective in this respect (Fig 3C). Given that CXCL12 mRNA expression was comparable in DA and AO non-immunized rats, in immunized DA rats it exceeded (p<0.001) that measured in their AO counterparts (Fig 3C).

Next, cell apoptosis within the DN thymocytes was examined. Immunization induced an increase (p<0.001) in the frequency of apoptotic cells within DN thymocytes in both rat strains, but this effect was particularly pronounced in DA rats (Fig 3D). Thus, although in non-immunized animals the frequency of apoptotic cells was higher (p<0.001) among DN thymocytes from AO than DA rats, in immunized rats it was lower (p<0.001) among these cells from AO rats (Fig 3D).

Irrespective of strain, immunization did not influence the frequency of proliferating cells among DN thymocytes compared with non-immunized strain-matched rats (Fig 3E). However, irrespective of immunization, higher (p<0.05) frequency of Ki-67+ cells was found among DN thymocytes from AO rats compared with DA ones (Fig 3E).

#### DP thymocyte subset composition

**Fig 4:**
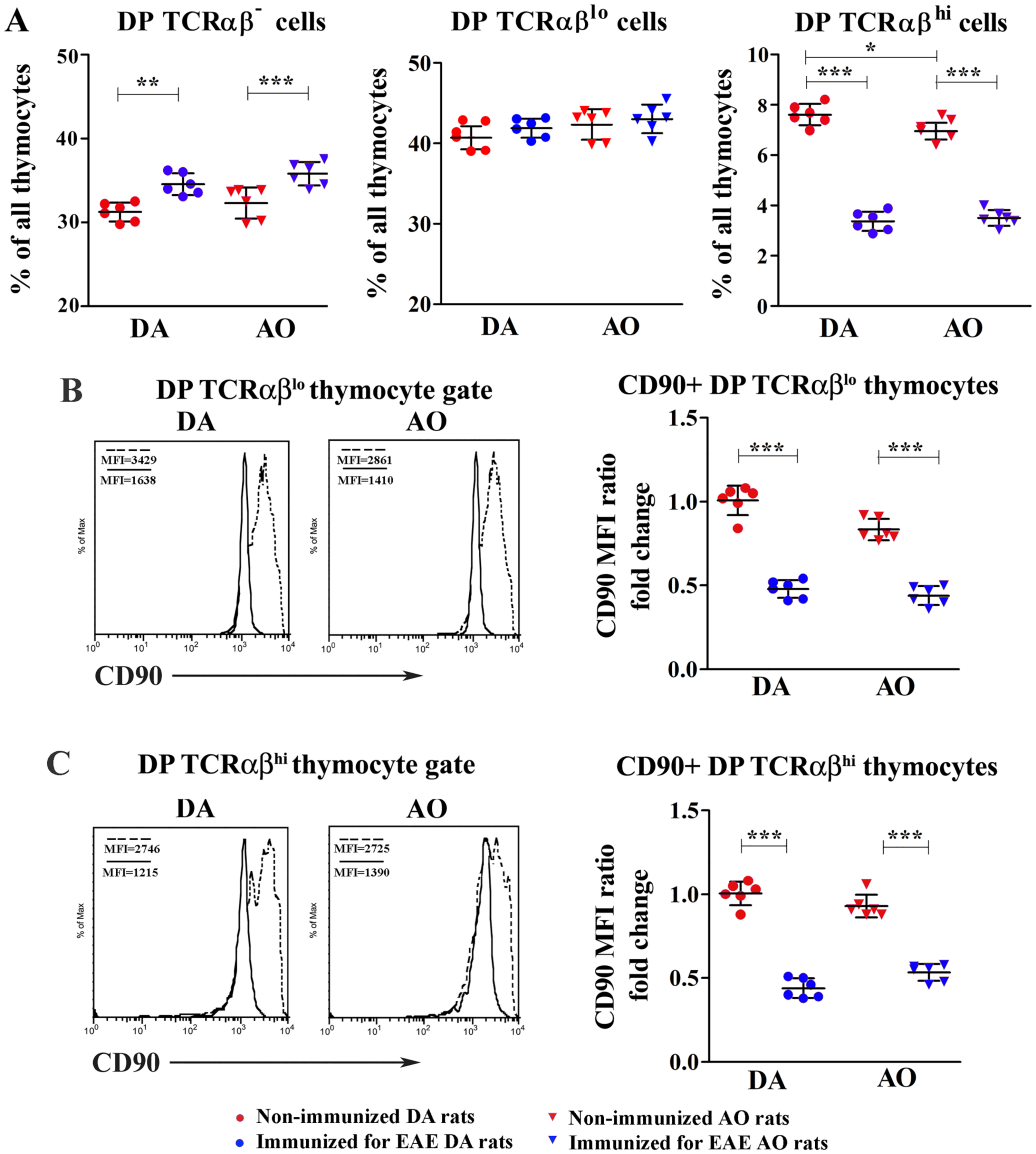
Effects of immunization for EAE on DP thymocyte phenotypic profile. **(A)** Scatter plots indicate the frequencies of CD4+CD8+ double positive (DP) thymocytes with non-detectable (TCRαβ^-^), low (TCRαβ^lo^) and high (TCRαβ^hi^) levels of TCRαβ surface expression among thymocytes from non-immunized and immunized for EAE DA and AO rats (gating strategy is displayed in S3A and S3B Fig). **(B,C)** Representative overlaid histograms indicate CD90 (Thy-1) surface expression on DP **(B)** TCRαβ^lo^ and **(C)** TCRαβ^hi^ thymocytes of (dashed line) non-immunized and (full line) immunized for EAE DA and AO rats. Data in the overlaid flow cytometry histograms are displayed as % of Max (the cell count in each bin divided by the cell count in the bin that contained the largest number of cells; http://www.flowjo.com), to allow visual comparison of samples with different event numbers collected. Scatter plots show the fold change in CD90 mean fluorescence intensity (MFI) ratio on CD90+ DP **(B)** TCRαβ^lo^ and **(C)** TCRαβ^hi^ thymocytes of non-immunized AO and immunized for EAE DA and AO rats, relative to non-immunized DA animals. Two way ANOVA showed significant interaction between the effects of strain and immunization for the frequency of DP TCRαβ^hi^ (F_(1,20)_ = 6.987, p<0.05) cells. Data points, means and ± SD are from one of two experiments with similar results (n = 6). * p<0.05; ** p<0.01; *** p<0.001.

Next, the phenotypic profile of DP thymocytes encompassing cells undergoing selection processes [33,34,56] was investigated. In both DA and AO rats immunization increased (p<0.01) the frequency of DP TCRαβ^-^ thymocytes which passed β selection [34] (Fig 4A). Irrespective of immunization, the frequency of these cells did not differ between DA and AO rats. The frequency of DP TCRαβ^lo^ thymocytes, entering positive selection processes [33,34], also increased in immunized rats of both strains, but this increase reached statistical significance in neither DA rats nor AO ones (Fig 4A). Thus, irrespective of immunization, their frequency was comparable in DA and AO rats (Fig 4A). On the other hand, the frequency of DP TCRαβ^hi^ thymocytes, corresponding to cells in an intermediate post-selection stage between DP TCRαβ^lo^ and SP TCRαβ^hi^ cells [33,34], markedly decreased (p<0.001) in immunized rats of both strains, but this decrease was more prominent in DA than in AO rats (Fig 4A). Consequently, as DP TCRαβ^hi^ cell frequency was lower (p<0.05) in AO than in DA non-immunized rats, following immunization their frequency did not differ between rats of these two strains (Fig 4A).

Considering that (i) immunization diminished the frequency of post-selected DP TCRαβ^hi^ cells and (ii) Thy-1 (CD90), which is highly expressed on the surface of DP thymocytes, negatively regulates TCR-mediated signaling and selection threshold during thymocyte differentiation in healthy rodents [58], its expression level on CD90+ DP thymocytes undergoing selection was examined. Irrespective of rat strain, the average surface density of Thy-1 expression (judging by MFI) on TCRαβ^lo^ and TCRαβ^hi^ CD90+ DP thymocytes was lower (p<0.001) in immunized rats than in strain-matched non-immunized rats (Fig 4B and 4C). Irrespective of immunization the average surface density of Thy-1 did not differ on either DP TCRαβ^lo^ or DP TCRαβ^hi^ thymocytes from DA and AO rats (Fig 4B and 4C).

#### CD4+ and CD8+ SP thymocyte subsets composition

**Fig 5:**
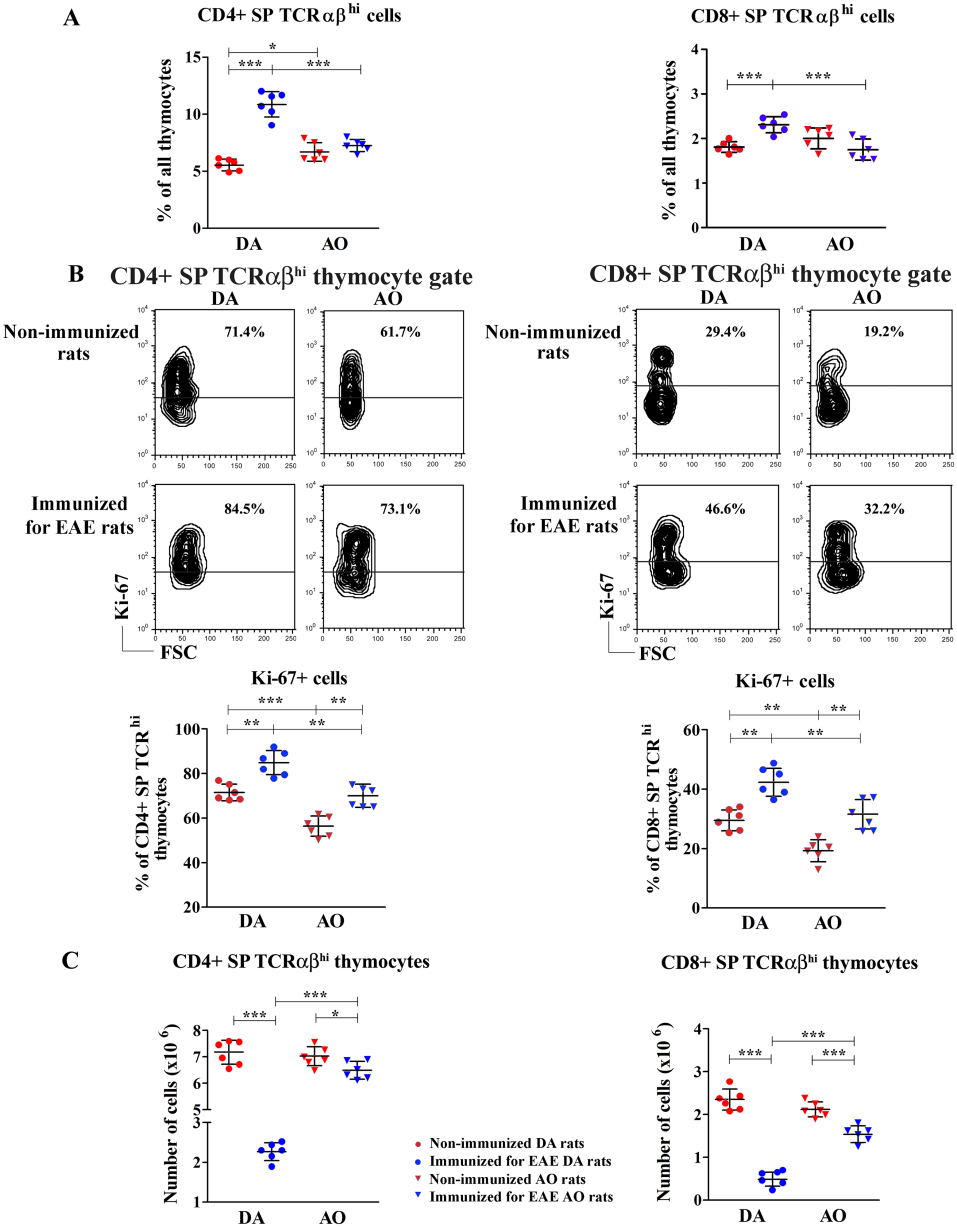
Immunization for EAE decreased the number of CD4+ and CD8+ SP TCRαβ^hi^ thymocytes more prominently in DA than in AO rats. **(A)** Scatter plots indicate the frequency of CD4+ and CD8+ TCRαβ^hi^ single positive (SP) thymocytes of non-immunized and immunized for EAE DA and AO rats (gating strategy is displayed in S3A and S3B Fig). **(B)** Representative flow cytometry contour plots show Ki-67 staining of CD4+ and CD8+ SP TCRαβ^hi^ thymocytes of non-immunized and immunized for EAE DA and AO rats (gating strategy is displayed in S2C and S2D Fig). Scatter plots indicate the frequency of Ki-67+ cells within CD4+ and CD8+ SP TCRαβ^hi^ thymocytes from non-immunized and immunized for EAE DA and AO rats. **(C)** Scatter plots indicate the number of CD4+ and CD8+ SP TCRαβ^hi^ thymocytes in non-immunized and immunized for EAE DA and AO rats. Two way ANOVA showed significant interaction between the effects of strain and immunization for the frequency of CD4+ (F_(1,20)_ = 55.12, p<0.001) and CD8+ SP TCRαβ^hi^ (F_(1,20)_ = 20.97, p<0.001) thymocytes, and the number of CD4+ (F_(1,20)_ = 229.8, p<0.001) and CD8+ SP TCRαβ^hi^ (F_(1,20)_ = 64.32, p<0.001) thymocytes. Data points, means and ± SD are from one of two experiments with similar results (n = 6). * p<0.05; ** p<0.01; *** p<0.001.

Next, the frequency of the most mature CD4+ and CD8+ SP cells, ready to leave the thymus was explored. Given that within both SP thymocyte subsets, apart from the most mature, TCRαβ^hi^ cells, immature SP (ISP) TCRαβ^-/lo^ cells were also identified [59,60] (S3C Fig), SP thymocytes were examined for the frequency of TCRαβ^hi^ cells. Immunization increased (p<0.001) the frequency of CD4+ and CD8+ SP TCRαβ^hi^ thymocytes in thymi from DA rats compared with the non-immunized strain-matched ones (Fig 5A). On the other hand, the frequency of CD4+ and CD8+ SP TCRαβ^hi^ cells remained unaltered in immunized AO rats compared with the non-immunized strain-matched ones (Fig 5A). In non-immunized animals, the frequency of CD4+ SP TCRαβ^hi^ thymocytes was slightly higher (p<0.05) in AO than in DA rats, whereas that of CD8+ SP TCRαβ^hi^ thymocytes did not differ between these two rat strains (Fig 5A). However, in immunized rats, the frequencies of the both SP TCRαβ^hi^ thymocyte subsets were lower (p<0.001) in AO rats than in their DA counterparts (Fig 5A).

To elucidate the mechanisms underlying the previous findings, the proliferation of SP TCRαβ^hi^ cells was analyzed. Irrespective of rat strain, immunization increased (p<0.01) the frequency of Ki-67+ proliferating cells among both CD4+ and CD8+ SP TCRαβ^hi^ thymocytes (Fig 5B). Additionally, irrespective of immunization, the frequency of proliferating Ki-67+ cells was lower (p<0.01) among CD4+ and CD8+ SP TCRαβ^hi^ thymocytes from AO rats when compared with DA ones (Fig 5B). Nevertheless, fewer CD4+ and CD8+ SP TCRαβ^hi^ thymocytes were retrieved from both immunized DA (p<0.001) and AO (p<0.05 and p<0.001 for CD4+ and CD8+ SP TCRαβ^hi^ thymocytes, respectively) rats compared with the strain-matched non-immunized ones (Fig 5C). These effects of immunization were more prominent in DA rats (Fig 5C). Consequently, although the numbers of both CD4+ and CD8+ SP TCRαβ^hi^ thymocytes were similar in DA and AO non-immunized rats, their numbers were lower (p<0.001) in immunized DA rats than in their AO counterparts (Fig 5C).

### Immunization for EAE prominently diminished the number of CD4+CD25+Foxp3+ thymocytes in DA rats

**Fig 6:**
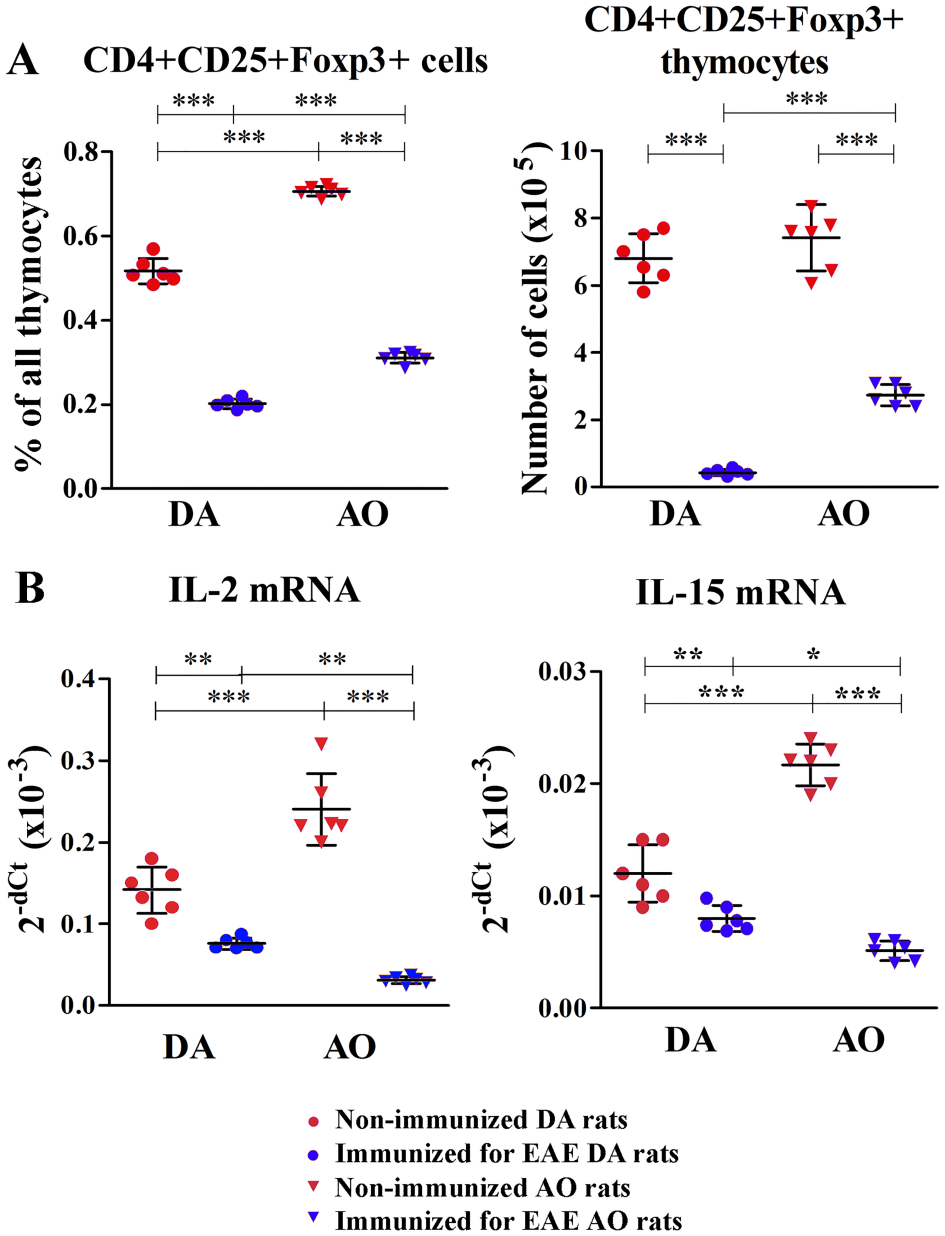
Immunization for EAE diminished the frequency and number of regulatory CD4+CD25+Foxp3+ thymocytes in DA more prominently than in AO rats. Scatter plots indicate **(A)** the frequency and the number of CD4+CD25+Foxp3+ thymocytes (gating strategy is displayed in S3A and S5 Figs) and **(B)** IL-2 and IL-15 mRNA expression in thymic tissue of non-immunized and immunized for EAE DA and AO rats. Two way ANOVA showed significant interaction between the effects of strain and immunization for the frequency (F_(1,20)_ = 152.2, p<0.001) and number (F_(1,20)_ = 7.296, p<0.05) of CD4+CD25+Foxp3+ thymocytes, and for thymic IL-2 (F_(1,20)_ = 28.31, p<0.001) and IL-15 (F_(1,20)_ = 64.0, p<0.001) mRNA expression. Data points, means and ± SD are from one of two experiments with similar results (n = 6). * p<0.05; ** p<0.01; *** p<0.001.

Next, intrathymic differentiation/maturation of CD4+ nTregs involved in the maintenance of self-tolerance and prevention of autoimmune disorders [61,62] was also examined. A dramatic drop (p<0.001) in the frequency of thymocytes expressing CD4+CD25+Foxp3+ phenotype of nTregs was found in rats of both strains immunized for EAE (Fig 6A). Similarly, the number of CD4+CD25+Foxp3+ thymocytes diminished (p<0.001) in immunized compared with strain-matched non-immunized rats (Fig 6A). Noteworthy, this drop was less prominent in AO compared with the corresponding DA rats. The frequency of CD4+CD25+Foxp3+ thymocytes was higher (p<0.001) in AO than in DA non-immunized rats, whereas their number was comparable between non-immunized rats of these two strains (Fig 6A). However, following immunization both the frequency and the number of CD4+CD25+Foxp3+ thymocytes were higher (p<0.001) in AO rats compared with DA ones (Fig 6A).

Next, considering that IL-2 and IL-15 are crucial for nTreg development [50], thymic expression of mRNA for these cytokines was examined. Immunization downregulated (p<0.01) the expression of both cytokines in both DA and AO rats (Fig 6B). The expression of mRNAs for IL-2 and IL-15 was greater (p<0.001) in AO than in DA non-immunized rats. On the contrary, immunized DA rats exhibited augmented (p<0.05) expression of these cytokines in respect to their AO counterparts (Fig 6B).

### Influence of immunization for EAE on the counts of CD4+ and CD8+ T-PBLs and their expression of CD28

#### Immunization for EAE decreased the counts of CD4+ and CD8+ T-PBLs in DA rats

**Fig 7:**
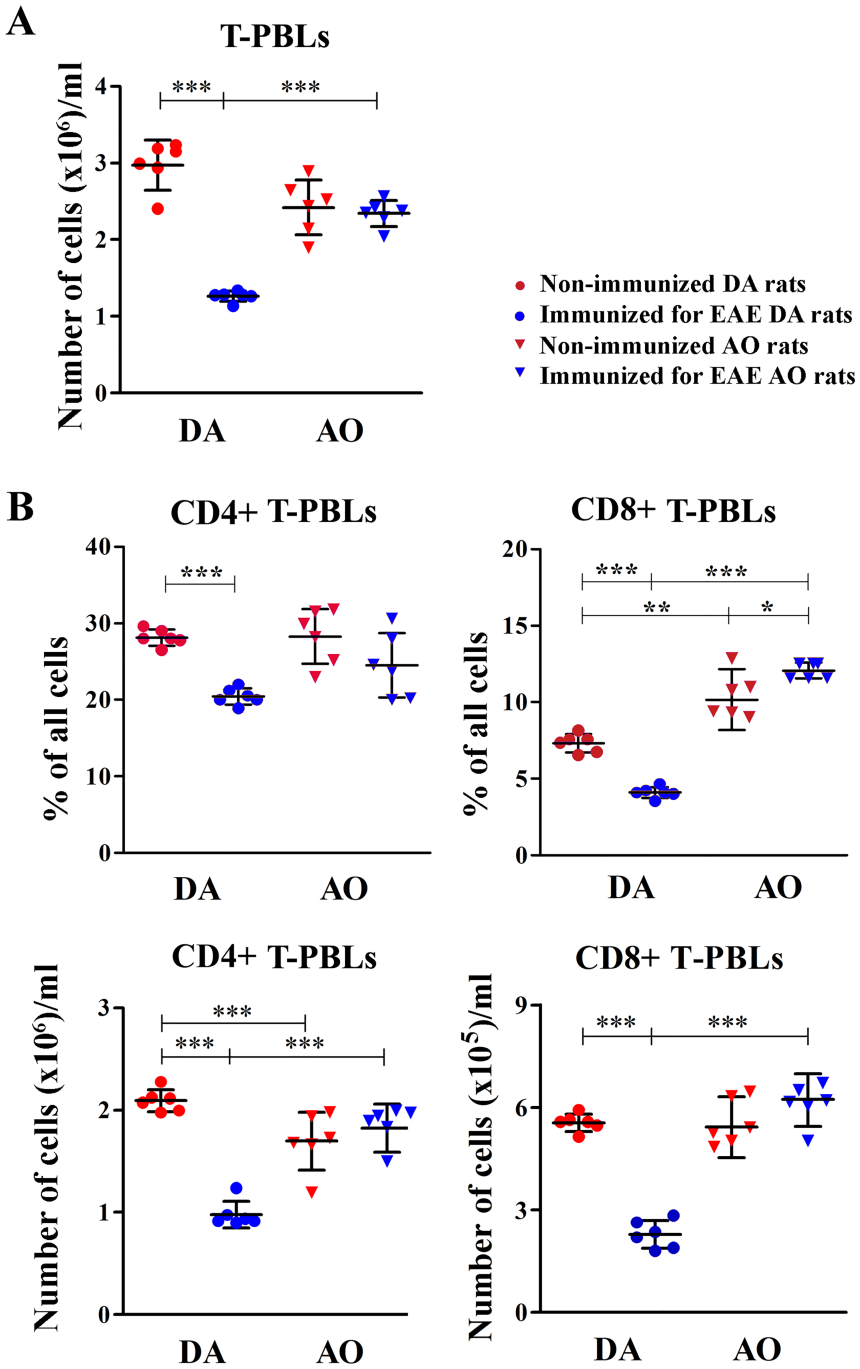
Immunization for EAE decreased the counts of CD4+ and CD8+ T-PBLs in DA rats. Scatter plots indicate **(A)** the number of T-peripheral blood lymphocytes (T-PBLs)/ml, and **(B)** (upper) the frequency and (lower) the number of CD4+ and CD8+ T-PBLs/ml retrieved from non-immunized and immunized for EAE DA and AO rats (gating strategy for T-PBLs and CD4+ and CD8+ T-PBLs is displayed in S6 Fig). Two way ANOVA showed significant interaction between the effects of strain and immunization for the frequency of CD8+ T-PBLs (F_(1,20)_ = 29.50, p<0.001), and the numbers of T-PBLs (F_(1,20)_ = 26.16, p<0.001), CD4+ (F_(1,20)_ = 69.30, p<0.001) and CD8+ (F_(1,20)_ = 193.9, p<0.001) T-PBLs. Data points, means and ± SD are from one of two experiments with similar results (n = 6). * p<0.05; ** p<0.01; *** p<0.001.

To evaluate the impact of the thymic changes on T-PBLs, these cells were examined for the frequency and count of CD4+ and CD8+ cells. Compared with the non-immunized rats, the number of T-PBLs diminished (p<0.001) only in immunized DA rats (Fig 7A). Consequently, although their number was similar between DA and AO non-immunized rats, it was markedly greater (p<0.001) in immunized AO rats than in their DA counterparts (Fig 7A). The frequency of CD4+ T-PBLs decreased (p<0.001) in immunized DA rats when compared with strain-matched non-immunized rats, whereas immunization did not affect their frequency in AO rats (Fig 7B). On the other hand, immunization decreased (p<0.001) and slightly increased (p<0.05) the frequency of CD8+ T-PBLs in DA and AO rats, respectively (Fig 7B). Consequently, the counts of both CD4+ and CD8+ T-PBLs diminished (p<0.001) in immunized DA rats compared with the strain-matched non-immunized ones (Fig 7B). On the other hand, compared with the strain-matched non-immunized rats, the counts of both CD4+ and CD8+ T-PBLs did not change significantly in immunized AO rats (Fig 7B). Irrespective of immunization, the frequency of CD4+ T-PBLs was comparable between DA and AO rats (Fig 7B). However, the CD4+ T-PBL count was diminished (p<0.001) in non-immunized AO rats compared with DA ones (Fig 7B). Differently, their count was greater (p<0.001) in immunized AO rats compared with their DA counterparts (Fig 7B). The frequency of CD8+ T-PBLs was higher in both non-immunized (p<0.01) and immunized (p<0.001) AO rats than in the corresponding DA ones (Fig 7B). However, strain difference in the CD8+ T-PBL count was found only in immunized rats, as more (p<0.001) CD8+ T-PBLs was recovered from AO than DA rats (Fig 7B).

#### Immunization for EAE decreased the frequency of CD90+CD45RC- RTEs, but increased that of CD90-CD45RC- memory phenotype cells among the major subpopulations of T-PBLs

**Fig 8:**
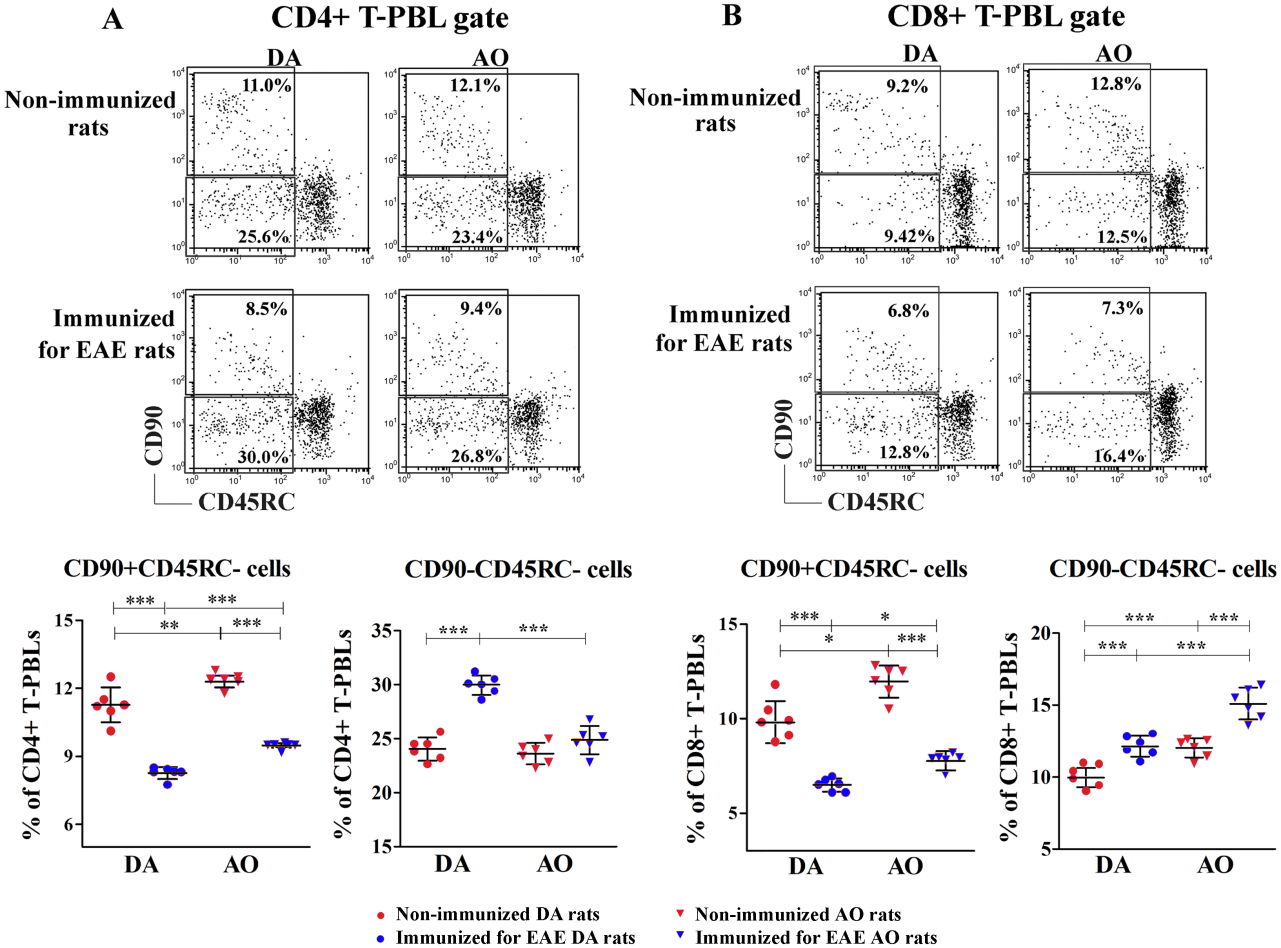
Immunization for EAE decreased the frequency of CD90+CD45RC-, but increased that of CD90-CD45RC- cells among the major subpopulations of T-PBLs from DA rats. Representative flow cytometry dot plots show CD90/CD45RC staining of **(A)** CD4+ and **(B)** CD8+ T-peripheral blood lymphocytes (T-PBLs) of non-immunized and immunized for EAE DA and AO rats (gating strategy for CD4+ and CD8+ T-PBLs is displayed in S6 Fig and for CD90+CD45RC- and CD90-CD45RC- cells in S7 Fig). Scatter plots indicate the frequency of CD90+CD45RC- cells (RTEs) and CD90-CD45RC- (memory phenotype) cells within (A) CD4+ and (B) CD8+ T-PBLs. Two way ANOVA showed a significant interaction between the effects of strain and immunization for the frequency of CD90-CD45RC- cells within CD4+ T-PBLs (F_(1,20)_ = 28.52, p<0.001). Data points, means and ± SD are from one of two experiments with similar results (n = 6). * p<0.05; ** p<0.01; *** p<0.001.

Considering immunization-related changes in thymopoiesis, the frequency of RTEs and cells with memory phenotype was also quantified. In the rat, RTEs are shown to have CD90+CD45RC- phenotype, whereas CD90-CD45RC- phenotype is suggested to correspond to that of memory cells [63]. Irrespective of strain, the frequency of RTEs among both CD4+ and CD8+ T-PBLs was lower (p<0.001) in immunized rats than in the strain-matched non-immunized ones (Fig 8A and 8B). This finding was suggestive of an erosion of the naïve T cell repertoire. Irrespective of immunization, the frequency of RTEs was higher (p<0.05) among the major T-PBL subpopulations from AO rats compared with DA ones (Fig 8A and 8B).

Next, considering that diminished thymopoiesis leads to expansion of certain T-cell clones, and consequently a relative increase in the pool of memory phenotype T cells [64,65], the frequency of CD90-CD45RC- cells among both CD4+ and CD8+ T-PBLs was examined. Their frequency among CD4+ T-PBLs was higher (p<0.001) in immunized DA rats compared with strain-matched non-immunized ones, but it did not significantly differ between non-immunized and immunized AO animals (Fig 8A). Consequently, the frequency of CD90-CD45RC- cells was comparable among CD4+ T-PBLs from non-immunized DA and AO rats, whereas it was lower (p<0.001) in immunized AO rats than in their DA counterparts (Fig 8A). On the other hand, compared with the strain-matched non-immunized rats, in immunized ones of both strains the frequency of CD90-CD45RC- cells increased (p<0.001) among CD8+ T-PBLs (Fig 8B). Irrespective of immunization, their frequency was higher (p<0.001) in AO rats compared with DA ones (Fig 8B).

**Fig 9:**
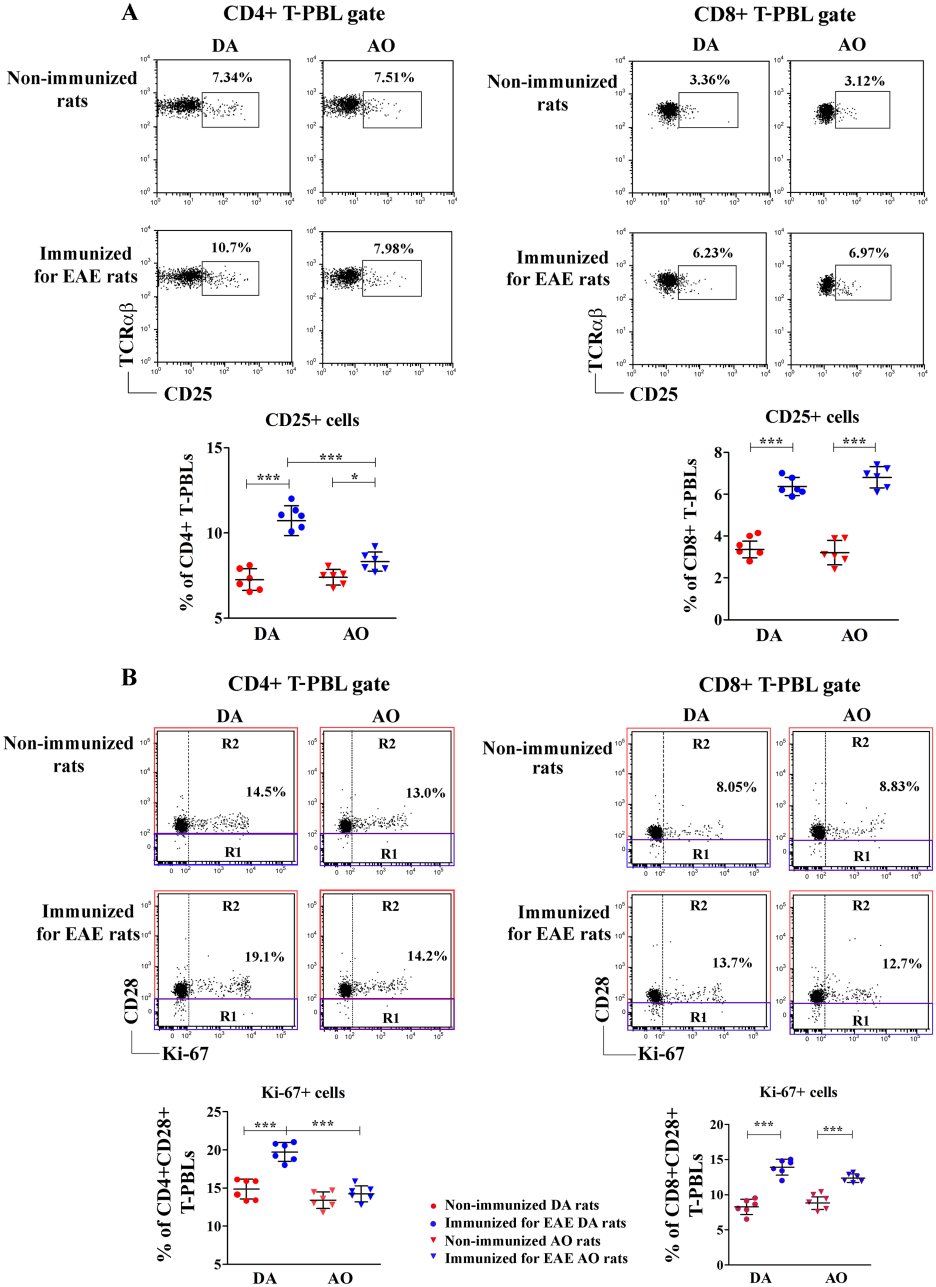
Immunization for EAE increased the frequency of activated and proliferating cells among the major subpopulations of T-PBLs. **(A)** Representative flow cytometry dot plots show CD25 expression on CD4+ and CD8+ T-peripheral blood lymphocytes (T-PBLs) from non-immunized and immunized for EAE DA and AO rats (gating strategy for CD4+ and CD8+ T-PBLs is displayed in S6 Fig). Scatter plots indicate the frequency of activated CD25+ cells among CD4+ and CD8+ T-PBLs from non-immunized and immunized for EAE DA and AO rats. **(B)** Representative flow cytometry dot plots show Ki-67 expression in CD4+CD28+ (R2) and CD8+CD28+ T-PBLs (R2) from non-immunized and immunized for EAE DA and AO rats. Please note that virtually there are no Ki-67+ cells among either CD4+CD28- (R1) or CD8+CD28- (R1) cells. Dashed line indicates cut off between Ki-67+ and Ki-67- cells. Scatter plots show the frequency of proliferating Ki-67+ cells among CD4+CD28+ and CD8+CD28+ T-PBLs from non-immunized and immunized for EAE DA and AO rats. Two way ANOVA showed significant interaction between the effects of strain and immunization for the frequency of CD25+ cells among CD4+ T-PBLs (F_(1,20)_ = 22.73, p<0.001), Ki-67+ cells among CD4+CD28+ (F_(1,20)_ = 30.04, p<0.001) and CD8+CD28+ (F_(1,20)_ = 7.182, p<0.05) T-PBLs. Data points, means and ± SD are from one of two experiments with similar results (n = 6). * p<0.05; *** p<0.001.

Considering that a decreased thymic output leads to compensatory T-cell activation and proliferation resulting in expansion of memory phenotype cell pool [7,8], the frequency of activated and Ki-67+ proliferating cells among the major subpopulations of T-PBLs was also examined. The frequency of activated CD25+ cells increased among CD4+ T-PBLs from immunized DA (p<0.001) and AO (p<0.05) rats compared with the strain-matched non-immunized ones, but this increase was more prominent in DA rats (Fig 9A). Consequently, although the frequency of CD25+ cells was similar among CD4+ T-PBLs from DA and AO non-immunized rats, following immunization their higher (p<0.001) frequency was registered in DA rats than in AO ones (Fig 9A). The frequency of CD25+ cells was also higher (p<0.001) among CD8+ T-PBLs from immunized rats of both strains when compared with the strain-matched non-immunized rats (Fig 9A). Irrespective of immunization, their frequency was comparable between the two rat strains (Fig 9A). The analysis of the frequency of activated CD69+ cells among CD4+ and CD8+ T-PBLs showed a similar pattern of differences to those described for the frequency of activated CD25+ cells (S8 Fig).

The analysis of the frequency of Ki-67+ cells among the major subpopulations of T-PBLs showed similar pattern of immunization-induced differences to those described in the frequency of activated CD25+ cells. Namely, their frequency was higher among CD4+CD28+ T-PBLs from immunized rats compared with the non-immunized strain-matched rats, but this difference reached statistical significance (p<0.001) only in DA rats (Fig 9B). Additionally, immunization increased (p<0.001) the frequency of Ki-67+ cells among CD8+CD28+ T-PBLs from both rat strains (Fig 9B). The frequencies of Ki-67+ cells among either CD4+CD28+ or CD8+CD28+ T-PBLs did not statistically significantly differ between DA and AO non-immunized rats (Fig 9B). On the other hand, their frequency among CD4+CD28+ T-PBLs was higher (p<0.001) in immunized DA rats compared with their AO counterparts, while it was comparable between CD8+CD28+ T-PBLs from DA and AO immunized rats (Fig 9B). Noteworthy, irrespective of rat strain Ki-67+ cells were extremely rare among CD4+CD28- and CD8+CD28- T-PBLs (Fig 9B).

#### Immunization for EAE increased the frequency of CD28- cells among CD4+ T-PBLs only in DA rats

**Fig 10:**
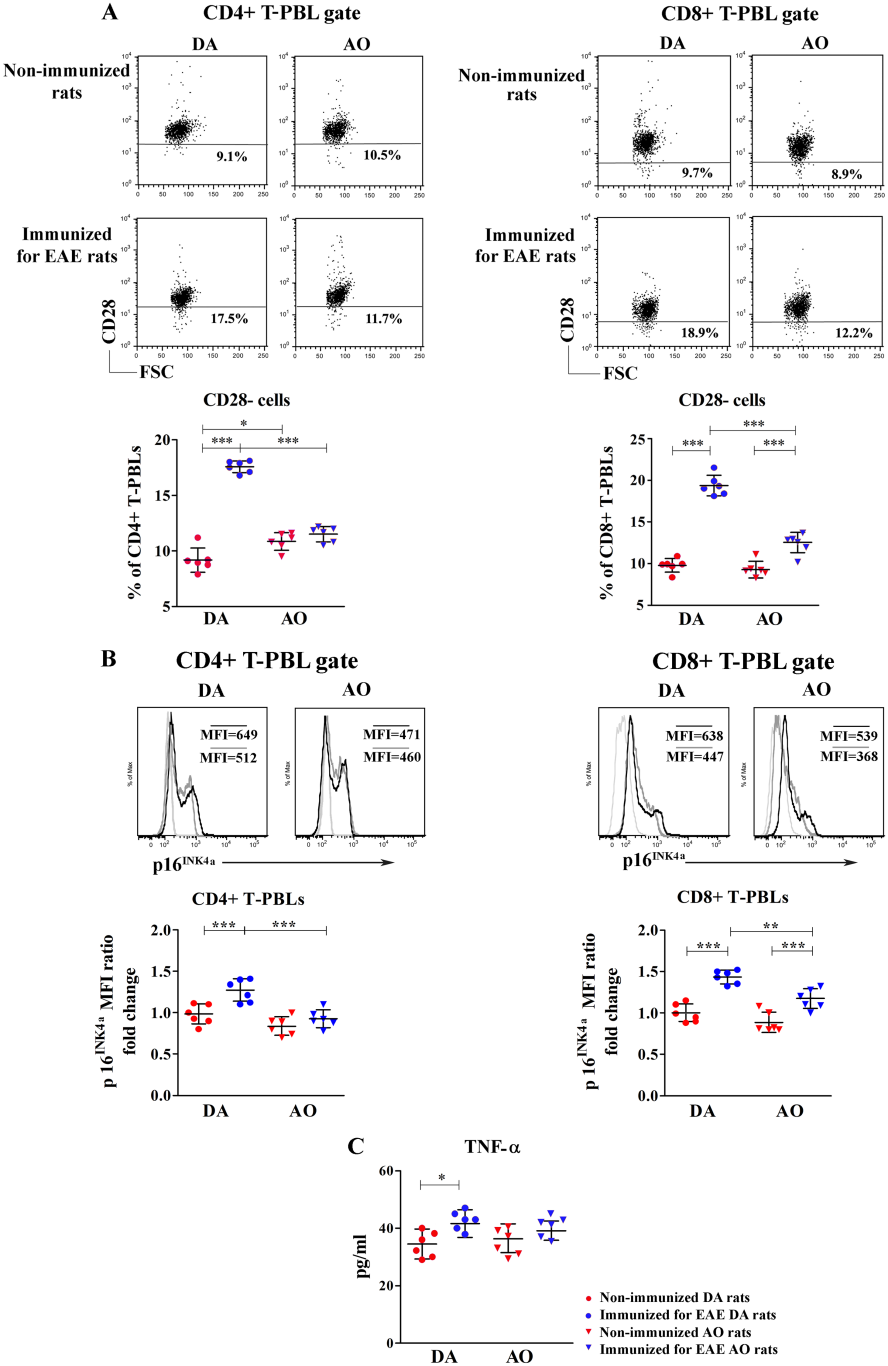
Immunization for EAE increased the frequency of CD28- cells among the major subpopulations of T-PBLs and elevated the circulating levels of TNF-α in DA rats. **(A)** Representative flow cytometry dot plots show CD28 staining of CD4+ and CD8+ T-peripheral blood lymphocytes (T-PBLs) from non-immunized and immunized for EAE DA and AO rats (gating strategy for CD4+ and CD8+ T-PBLs is displayed in S6 Fig and for CD28- cells among them in S9 Fig). Scatter plots indicate the frequency of CD28- cells among CD4+ and CD8+ T-PBLs from non-immunized and immunized for EAE DA and AO rats. **(B)** Representative overlaid flow cytometry histograms show p16^INK4a^ expression in CD4+ and CD8+ T-PBLs from non-immunized (dark gray line) and immunized for EAE (black line) DA and AO rats. Scatter plots indicate the fold change in p16^INK4a^ mean fluorescence intensity (MFI) in CD4+ and CD8+ T-PBLs of non-immunized AO and immunized for EAE DA and AO rats, relative to non-immunized DA animals. Data in the overlaid flow cytometry histograms are displayed as % of Max (the cell count in each bin divided by the cell count in the bin that contained the largest number of cells; http://www.flowjo.com), to allow visual comparison of samples with different event numbers collected. **(C)** Scatter plot indicates the concentration of TNF-α in plasma of non-immunized and immunized for EAE DA and AO rats. Two way ANOVA showed significant interaction between the effects of strain and immunization for the frequency of CD28- among CD4+ (F_(1,20)_ = 125.8, p<0.001) and CD8+ (F_(1,20)_ = 44.12, p<0.001) T-PBLs. Data points, means and ± SD are from one of two experiments with similar results (n = 6). * p<0.05; ** p<0.001; *** p<0.001.

Given that the decrease in the thymic T-cell output leads to their homeostatic oligoclonal proliferation [7,8] and loss of CD28 surface expression [66], the frequency of CD28- cells among CD4+ and CD8+ T-PBLs was also determined. Immunization for EAE increased (p<0.001) the frequency of CD28- cells among CD4+ T-PBLs in DA rats, while their frequency remained unaltered in AO rats (Fig 10A). Accordingly, the frequency of CD28- cells was higher (p<0.05) among CD4+ T-PBLs from non-immunized AO compared with DA rats (Fig 10A). On the contrary, in immunized rats it was lower (p<0.001) in AO than in DA rats (Fig 10A). The proportion of CD28- cells among CD8+ T-PBLs markedly increased (p<0.001) in immunized rats of both strains when compared with the strain-matched non-immunized animals, but this increase was more prominent in DA rats (Fig 10A). Consequently, although their frequency did not significantly differ between DA and AO non-immunized rats, it was higher (p<0.001) in immunized DA animals than in their AO counterparts (Fig 10A).

Next, considering that (i) CD28- cells exhibit replicative exhaustion [25], and (ii) p16^INK4a^, a biomarker of replicative senescence, accumulates in cells undergoing replicative exhaustion during inflammation/infection [67], the average amount of p16^INK4a^ per T-PBL (judging by MFI) was determined. In accordance with the changes in the frequency of CD28- cells, accumulation of p16^INK4a^ (p<0.001) was detected in CD4+ T-PBLs from immunized DA rats and CD8+ T-PBLs from immunized DA and AO rats in respect to the strain-matched non-immunized ones (Fig 10B). The expression level of p16^INK4a^ was comparable in CD4+ and CD8+ T-PBLs from DA and AO non-immunized rats (Fig 10B). However, in immunized rats its expression level was lower (p<0.01) in both CD4+ and CD8+ T-PBLs from AO rats compared with DA ones (Fig 10B).

Considering that the development of inflammatory autoimmune diseases is associated with increased circulating TNF-α levels and that this cytokine directly downregulates CD28 expression on CD4+ T cells [23,24,52], the level of circulating TNF-α was also determined. Consistent with the increased frequency of CD28- cells within CD4+ T-PBLs of immunized DA rats, the circulating level of TNF-α in these animals increased (p<0.05) upon immunization, while it remained unaltered in AO rats (Fig 10C). Thus, although the circulating TNF-α level was comparable between non-immunized DA and AO rats, it was slightly higher in immunized DA rats compared with their AO counterparts, but this increase did not reach statistical significance (Fig 10C).

### Greater frequency of granzyme B-producing CD4+ T lymphocytes in SC of DA rats with clinical EAE than in immunized AO rats

**Fig 11:**
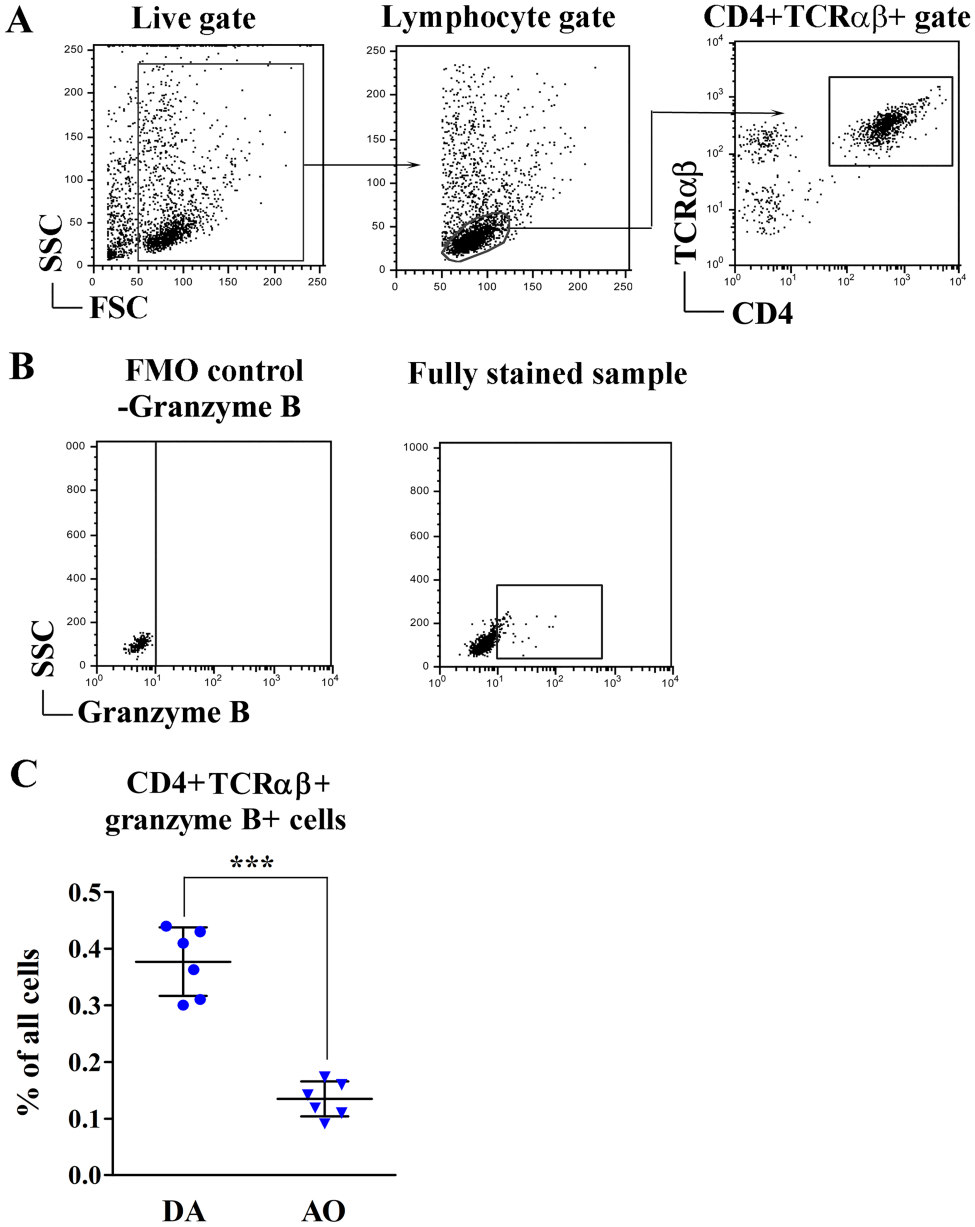
Greater frequency of granzyme B+ CD4+ T cells in SC mononuclear infiltrate from DA rats immunized for EAE than in their AO counterparts. Flow cytometry dot plots show **(A)** gating strategy for CD4+TCRαβ+ lymphocytes, **(B)** fluorescence minus one (FMO) control staining for setting cut off boundary for analysis of granzyme B expression in CD4+TCRαβ+ lymphocytes gated as shown in **(A)**, and granzyme B expression in CD4+TCRαβ+ lymphocytes (fully stained sample). **(C)** Scatter plot indicates the frequency of granzyme B+ CD4+ T cells in mononuclear cell infiltrate retrieved from spinal cord (SC) of DA and AO rats immunized for EAE. Student’s *t*-test was used to assess the statistical significance of differences between DA and AO rats. Data points, means and ± SD are from one of two experiments with similar results (n = 6). *** p<0.001.

Given that cytolytic granzyme B-producing CD4+CD28- cells are shown to migrate to sites of inflammation in MS where they contribute to tissue damage so that their greater frequency correlates with worse clinical outcome [24,44,68], the frequency of granzyme B+ CD4+ T cells infiltrating SC of rats immunized for EAE was examined. Their frequency was considerably higher (p<0.001) in SC of DA rats when compared with their AO counterparts (Fig 11). Irrespective of strain, their frequency in the non-immunized rats was negligible.

## Discussion

The study complements our previous research exploring strain differences in peripheral and target-tissue immune responses to immunization for EAE [42,69] by providing insight into immunization-induced changes in thymopoiesis. It showed that immunization with syngeneic SC homogenate in CFA leads to different changes in the thymus and the peripheral T-cell compartment in DA rats developing clinical signs of EAE, and in AO rats, which did not develop clinically manifested EAE.

### More prominent influence of immunization for EAE on thymic atrophy in DA rats compared with AO ones

The herein reported findings, consistent with some previous studies [27,28], showed a pronounced decrease in the thymic weight and thymocyte yield in rats developing clinically manifested EAE. On the other hand, only a modest thymic atrophy and decrease in the thymocyte yield were found in AO rats which failed to develop clinical signs of EAE. The decreased normalized thymic weight and thymocyte yield in DA rats immunized for EAE indicated a thymus-specific atrophic effect of immunization. It is noteworthy that thymic atrophy was also observed in MS and suggested to be indicative of “premature immunosenescence” [17,21,22]. In our study, the thymic atrophy in rats of both strains was partly linked with increased thymocyte apoptosis, which was particularly prominent in DA rats. Augmented apoptotic elimination of thymocytes is also observed in other experimental models of autoimmunity [70,71], and in aging rodents [38,72]. Given that IL-7 upregulates the expression of Bcl-2 family of antiapoptotic proteins and promotes survival of developing T cells in the thymus [46,47], the enhanced thymocyte apoptosis in the immunized rats could be linked with the downregulation of its expression. To corroborate this assumption are data indicating that early thymic involution in rapid-involuting strains of mice is linked with a premature downregulation of IL-7 expression in thymic cortical epithelial cells, and consequently, down- and upregulation of thymocyte anti-apoptotic Bcl-2 and pro-apoptotic BAD gene expression, respectively [73]. The changes in IL-7 expression are consistent with the increased circulating levels of IL-6 and enhanced thymic IL-6 mRNA expression in DA rats. Namely, it was shown that an increase in circulating IL-6 levels and/or thymic IL-6 expression leads to thymic atrophy due to direct thymic epithelial cell damage with preferential loss of cortical epithelium and decrease in DP thymocyte number [49,74,75]. Additionally, given that: i) in an acute inflammatory syndrome TNF-α induces thymic atrophy and accelerated thymocyte apoptosis [51], and ii) TNF-α is shown to upregulate thymic IL-6 expression [75], the thymic alterations in the immunized DA rats could also be associated with the increased circulating levels of TNF-α.

### Effects of immunization for EAE on thymopoiesis were more pronounced in DA rats than in AO ones

Immunization for EAE affected thymocyte subset distribution in rats of both strains. It increased the frequency of the most immature DN TCRαβ^-^ thymocytes in rats of both strains, but this effect was more prominent in DA rats. This increase in DA rats could reflect: i) a reduced apoptotic elimination of DN thymocytes, ii) increased proliferation of DN thymocytes, iii) decreased β selection and/or transitional block to the DP maturational stage and iv) increased ingress of T-cell precursors into the thymus. The rise in the frequency of apoptotic cells among DN thymocytes from these animals did not support the first option. Given that immature thymocytes are exceptionally sensitive to the lack of IL-7 [76], a causal relation between the decrease in the amount of the IL-7 transcript in the thymus and the augmented apoptosis of DN thymocytes in DA rats may be assumed. A contribution of an augmented cell proliferation to the rise in the frequency of DN thymocytes did not seem likely, as we failed to show any significant influence of immunization on their proliferation. The increased proportion of the descendant DP TCRαβ^-^ cells encompassing mostly cells that have surpassed β selection [34] dismissed the possibility of maturational block at the DN TCRαβ^-^ developmental stage. Consequently, it seems apparent that the rise in the frequency of the most immature DN cells in the thymus, at least in DA rats with EAE, reflected an enhanced ingress of their extrathymic precursors. To support this notion were the increased frequency of the most immature CD45RC+CD2-/+ DN cells among thymocytes from DA rats immunized for EAE, and the upregulated thymic CXCL12 expression compared with the strain-matched non-immunized rats. Namely, it is shown that: i) CXCL12 is critical for recruitment and homing of lymphoid progenitors in the thymus [45,77], and ii) despite the prominent thymic involution its expression is strong in aged humans [75]. In favor of the previous assumption are also data indicating that emptying of the thymic niches followed by a decreased thymic T-cell export and contraction of the peripheral T-lymphocyte pool provides feedback signals promoting increased progenitor entry [78]. In this respect the absence of prominent changes in the thymic CXCL12 expression in AO rats exhibiting milder thymic atrophy did not seem to be surprising.

Analysis of the downstream thymocyte maturational steps in rats of both strains immunized for EAE showed that the increase in the frequency of DP TCRαβ^-^ thymocytes was followed by unaltered frequency of DP TCRαβ^lo^ thymocytes entering selection [33,34] and diminished frequency of DP TCRαβ^hi^ thymocytes that surpassed positive selection [34]. These findings were suggestive of a less efficient positive selection and/or an excessive negative selection in rats immunized for EAE. In favor of this assumption was the reduced surface density of Thy-1 on TCRαβ-expressing subsets of DP thymocytes from rats of both strains immunized for EAE. Namely, it is shown that thymocytes from Thy-1^-/-^ mice exhibit augmented negative selection due to a lack of Thy-1-mediated negative regulation of TCRαβ signaling and selection threshold during thymocyte differentiation/maturation [58]. Additionally, to corroborate this notion are data indicating aging-related decrease of Thy-1 expression on the surface of rat DP TCRαβ^lo^ thymocytes followed by a decline in the frequency of post-selected DP TCRαβ^hi^ thymocytes [38]. Given that catecholamines were shown to downregulate Thy-1 mRNA expression [79], the decreased surface density of Thy-1 in immunized rats could be also related to data indicating an increase in the circulating levels of catecholamines during active EAE and MS [80].

Despite the reduced frequency of their DP TCRαβ^hi^ ancestors in immunized rats of both strains, the frequencies of the most mature CD4+ and CD8+ SP TCRαβ^hi^ thymocytes increased in DA rats, while they remained unaltered in AO animals compared with the corresponding non-immunized rats. In light of data indicating enhanced intrathymic differentiation/maturation of T cells beyond the developmental “blocks” [81], the previous findings suggested a more efficient differentiation of the positively selected cells towards SP TCRαβ^hi^ cells. The increase in the frequency of CD4+ SP TCRαβ^hi^ thymocytes in DA rats could also be explained by the positive feedback effects of mature CD4+ SP cells on DP thymocyte differentiation towards the CD4+ lineage [82]. Furthermore, the increased/unaltered frequency of the most mature SP TCRαβ^hi^ thymocytes could also suggest their increased premigrational expansion [83]. In favor of this notion is the greater frequency of proliferating cells among CD4+ and CD8+ SP TCRαβ^hi^ thymocytes in immunized rats compared with the strain-matched non-immunized ones. However, despite the increased/unaltered frequency of these cells in the immunized compared with the strain-matched non-immunized rats, the number of SP TCRαβ^hi^ thymocytes diminished in immunized rats of both strains suggesting that the compensatory mechanisms were not sufficient to overcome the thymopoietic defects. It should be pointed out that the thymic changes were less prominent in AO rats, as shown by the markedly more pronounced decrease in the number of the most mature CD4+ and CD8+ SP TCRαβ^hi^ thymocytes in DA rats compared with AO ones.

Given that (i) in immunized rats the decline in CD4+CD25+Foxp3+ thymocyte number was more prominent than that in the number of all mature SP CD4+ cells and (ii) this decline was more pronounced in immunized DA rats than in their AO counterparts, it may be assumed that the differentiation/maturation of nTregs was particularly affected in the inflammatory autoimmune pathology. Our results were at odds to those obtained in MOG-induced mouse EAE model [84]. This inconsistency could be related to data indicating that the thymic changes in EAE models are antigen-specific [84]. Considering that the common γ-chain receptor-dependent cytokines produced by thymocytes (IL-2) and thymic stromal cells (IL-7, IL-15) are crucial for nTreg development [48,50], the decline in the frequency of CD4+CD25+Foxp3+ thymocytes in rats immunized for EAE most likely reflected the decline in their expression. The reduced IL-2 expression in thymi from the immunized rats is consistent with data indicating that (i) recirculating Tregs inhibit the differentiation of their precursors by limiting the availability of IL-2 and (ii) the proportion of recirculating Tregs among thymocytes grows substantially with age, infection, stress, resulting in a strongly fading *de novo* development of Tregs [50]. The more pronounced decrease of the number of nTregs in DA rats, despite the smaller decrease in the expression of IL-2 and IL-15 compared with the EAE-resistant AO rats, could be related to data showing impaired thymic expression of STAT5, a downstream molecule in their signaling [85], in mice developing clinically manifested EAE [86]. The dramatic drop in the number of nTregs in DA rats also correlated with the severe neurological deficit found in these animals. This is consistent with data indicating that: i) thymectomy inhibits spontaneous remission in rat EAE [87] and ii) newly generated nTregs are critical for Treg-mediated autoimmune response suppression in MS [88]. Finally, it is noteworthy that: i) the overall reduction in thymic T-cell output with aging is also accompanied by diminished production of nTregs [89] and ii) this is particularly prominent in “premature aging” [17,18].

### Immunization for EAE decreased the frequency of CD4+ and CD8+ RTEs in DA and AO rats and led to accumulation of CD4+CD28- cells in DA rats

In accordance with some previous studies [90], our findings showed a marked decrease in the counts of both CD4+ and CD8+ T-PBLs in DA rats, but not in AO rats. This decrease in the T-PBL count in DA rats could reflect an enhanced migration of T cells, particularly CD4+ T cells into the CNS [90,91] and/or immunization-induced thymic atrophy and impaired output of naïve T cells. In favor of the latter was the reduced frequency of RTEs in both CD4+ and CD8+ T-PBL subpopulations from immunized DA rats compared with the strain-matched non-immunized ones. This is consistent with data indicating that in MS patients the levels of circulating CD4+ and CD8+ T-PBLs expressing TCR excision circles (TRECs), traceable molecular markers that identify RTEs, decrease with acute relapses to match those of 30 years older healthy controls [92]. The age-associated decline in RTEs is associated with propensity for development of autoimmunity [93]. Impaired thymopoiesis is shown to be accompanied by homeostatic proliferation of peripheral T cells [7,8]. The most acknowledged phenotypic change of lymphocytes undergoing such replicative stress in humans is the loss of surface CD28 expression on T cells, which is particularly prominent in CD8+ ones [66]. The frequency of CD28- cells increased among CD8+ T-PBLs from immunized rats of both strains, whereas the frequency of CD28- cells increased only among CD4+ T-PBLs from immunized DA rats. An accumulating body of evidence indicates loss of CD28 expression on the surface of CD4+ T cells from patients with inflammatory autoimmune diseases [23,24,44]. This CD28 loss in DA rats could be partly associated with CD4+ T-cell replicative stress evidenced by (i) the increased frequency of activated/proliferating cells among them [7,8], (ii) the expansion of the memory phenotype CD4+ cell pool [64,65] and iii) the accumulation of p16^INK4a^, a replicative senescence marker, within CD4+ T cells [67]. Additionally, the rise in TNF-α level in immunized DA rats could also contribute to this phenomenon [23,24,52]. Given that synthesis of granzyme B is a hallmark of CD4+CD28- T cells [44], the greater frequency of CD4+granzyme B+ cells in the SC of immunized DA rats compared with AO ones further corroborates their greater generation in DA rats. Considering that a greater frequency of cytolytic CD4+CD28- T cells in peripheral blood of MS patients is associated with a worse clinical outcome [44], while a greater frequency of CD4+granzyme B+ cells in SC of mice is associated with a more pronounced tissue damage [94], their higher frequency in the SC of DA rats compared with AO ones correlated with the more severe neuroinflammation in DA animals. It is noteworthy that the loss of CD28 transcription in inflammatory pathologies is reversible, which could be of therapeutic relevance [23]. Thus, the increase in the frequency of peripheral blood CD4+CD28- T cells is suggested to be not only a biomarker of “premature immunesenesence” in inflammatory autoimmune pathologies, but also a prognostic factor in these pathologies [23,24]. On the other hand, CD8+CD28- T cells represent heterogeneous cell population consisting of cells with proinflammatory and cytolytic characteristics, but also of those exhibiting regulatory properties [95,96]. Thus, the consequences of the rise in their frequency for autoimmune disease pathogenesis are not easy to predict.

In conclusion, the study provided additional evidence for the premature decline in thymopoiesis and appearance of phenomena related to immune senescence in the peripheral T-cell compartment (viz. the decreased frequency of RTEs followed by an increase in the frequency of CD28- cells among T-PBLs and expansion of memory T-cell pool) during the development of clinically manifested EAE in DA rats. Additionally, it pointed out to a prominent decline in thymic generation of CD4+ nTregs in the immunized rats. Thus, it suggested that the thymic changes occurring over the course of autoimmune diseases might not only leave the host vulnerable to infections, malignity and/or decreased vaccinal response due to a narrowing of the peripheral naïve T cell repertoire, but also contribute to perpetuation of the disease by impairing the generation of nTregs and favoring a generation of CD4+CD28- T cells. In addition, the study points to a number of cellular and molecular mechanisms underlying the thymic changes ultimately leading to the decline in thymic T-cell output in rodent models of CD4+ T cell-mediated autoimmune diseases, and suggests the significance of animal genetic background for these changes. Thus, the study as a whole, apart from fundamental importance, may be relevant for designing new therapeutic approaches to autoimmune diseases, which will take into consideration individual immunological abnormalities at various levels, including the thymus.

## Supporting information

S1 Fig Differently from AO rats, DA rats immunized for EAE develop clinically manifested disease. **(A)** Line graph indicates the mean daily clinical score of experimental autoimmune encephalomyelitis (EAE) in DA and AO rats immunized with rat spinal cord homogenate supplemented with complete Freund’s adjuvant, from the day of EAE onset to the 13^th^ day post immunization (d.p.i.), which correponds to the peak of the disease in DA rats. Scatter plots indicate **(A)** the maximal neurological score of DA and AO rats immunized for EAE and **(B)** the body weight (BW) of DA and AO rats immunized for EAE recorded on the day of immunization and on the 13^th^ d.p.i. Data points, means and ± SD are from one experiment of two sets of experiments with similar results (n = 6). * p<0.05.
(TIF)

S2 Fig Gating strategy for flow cytometry analysis of Ki-67 staining of thymocytes and distinct thymocyte subsets. Gating strategy based on fluorescence minus one (FMO) controls for setting cut off boundaries for analysis of Ki-67 expression in Ki-67/TCRαβ/CD4/CD8 stained thymocytes from non-immunized and immunized for EAE DA and AO rats. Flow cytometry contour plots represent FMO controls without anti-Ki-67 mAb and corresponding fully stained cells within **(A)** thymocytes, **(B)** CD4-CD8- double negative (DN), **(C)** CD4+ single positive (SP) TCRαβ^hi^ and **(D)** CD8+ SP TCRαβ^hi^ thymocyte gate.
(TIF)

S3 Fig Gating strategy for flow cytometry analysis of TCRαβ/CD4/CD8 staining of thymocytes. **(A)** Flow cytometry dot plots represent fluorescence minus one (FMO) controls without anti-CD4 or anti-CD8 mAbs and fully stained thymocytes (gated within the live gate, as shown on the appropriate flow cytometry dot plots). R1 = CD4-CD8- (double negative, DN) thymocytes; R2 = CD4+CD8+ (double positive, DP) thymocytes; R3 = CD4+ (single positive, SP) thymocytes and R4 = CD8+ SP thymocytes. **(B)** Flow cytometry histograms represent FMO control without anti-TCRαβ mAb and fully stained thymocytes. **(C)** Representative flow cytometry histograms show TCRαβ expression on DN, DP, CD4+ and CD8+ SP thymocytes (gated as shown in A) of non-immunized and immunized for EAE DA and AO rats.
(TIF)

S4 Fig Gating strategy for flow cytometry analysis of CD4/CD8/CD2/CD45RC staining of thymocytes. Gating strategy based on fluorescence minus one (FMO) controls for setting cut off boundaries for analysis of CD2/CD45RC expression on CD4/CD8 stained thymocytes from non-immunized and immunized for EAE DA and AO rats. Flow cytometry dot plots represent FMO controls without anti-CD2 or anti-CD45RC mAbs and fully stained cells within the CD4-CD8- double negative (DN) thymocyte gate (gated as shown in S3A Fig). R1 = CD45RC+CD2- DN thymocytes; R2 = CD45RC+CD2+ DN thymocytes.
(TIF)

S5 Fig Gating strategy for flow cytometry analysis of CD4/CD25/Foxp3 staining of thymocytes. Gating strategy based on fluorescence minus one (FMO) controls for setting cut off boundaries for analysis of CD4/CD25/Foxp3 expression on thymocytes from non-immunized and immunized for EAE DA and AO rats. Flow cytometry dot plots represent FMO controls without anti-CD25 or anti-Foxp3 mAbs and fully stained cells within the CD4+ SP thymocyte gate (gated as shown in S3A Fig).
(TIF)

S6 Fig Gating strategy for flow cytometry analysis of TCRαβ/CD4/CD8 staining of peripheral blood lymphocytes. Gating strategy for analysis of TCRαβ/CD4/CD8 stained T-peripheral blood lymphocytes (T-PBLs) from non-immunized and immunized for EAE DA and AO rats. CD4 and CD8 expression was analyzed in T-PBLs (TCRαβ+ cells), gated as shown on the flow cytometry histogram. TCRαβ+ cells were gated within live lymphocytes, as shown on the appropriate flow cytometry dot plots.
(TIF)

S7 Fig Gating strategy for flow cytometry analysis of TCRαβ/CD4/CD90/CD45RC and TCRαβ/CD8/CD90/CD45RC staining of peripheral blood lymphocytes. Gating strategy based on fluorescence minus one (FMO) controls for setting cut off boundaries for analysis of CD90/CD45RC expression on CD4+ and CD8+ T-peripheral blood lymphocytes (T-PBLs) from non-immunized and immunized for EAE DA and AO rats. Flow cytometry dot plots represent FMO controls without anti-CD90 or anti-CD45RC mAbs and fully stained cells within **(A)** CD4+ and **(B)** CD8+ T-PBLs (gating strategies for CD4+ and CD8+ T-PBLs are displayed in S6 Fig). R1 = CD45RC-CD90+ cells (RTEs); R2 = CD45RC-CD90- cells (memory phenotype).
(TIF)

S8 Fig Gating strategy and analysis of CD69 expression on CD4+ and CD8+ T-PBLs. **(A,B)** Gating strategy based on fluorescence minus one (FMO) controls for setting cut off boundaries for analysis of CD69 expression on CD4+ and CD8+ T-peripheral blood lymphocytes (T-PBLs) from non-immunized and immunized for EAE DA and AO rats. Flow cytometry dot plots represent FMO controls without anti-CD69 Ab and fully stained cells within **(A)** CD4+ and **(B)** CD8+ T-PBLs (gating strategies for CD4+ and CD8+ T-PBLs are displayed in S6 Fig). **(C)** Flow cytometry dot plots show CD69 staining of CD4+ and CD8+ T-PBLs of non-immunized and immunized for EAE DA and AO rats. Scatter plots indicate the frequency of CD69+ cells within CD4+ and CD8+ T-PBLs. Two way ANOVA showed significant interaction between the effect of strain and immunization for the frequency of CD69+ cells within CD4+ T-PBLs (F_(1,20)_ = 56.89, p<0.001). Data points, means and ± SD are from one of two experiments with similar results (n = 6). * p<0.05; ** p<0.01; *** p<0.001.
(TIF)

S9 Fig Gating strategy flow cytometry analysis of TCRαβ/CD4/CD28 and TCRαβ/CD8/CD28 staining of T-PBLs. Gating strategy based on fluorescence minus one (FMO) controls for setting cut off boundaries for analysis of CD28 expression on **(A**) TCRαβ/CD4 and **(B)** TCRαβ/CD8 stained T-peripheral blood lymphocytes (T-PBLs) from non-immunized and immunized for EAE DA and AO rats (gating strategies for CD4+ and CD8+ T-PBLs are displayed in S6 Fig). Flow cytometry dot plots represent FMO controls without anti-CD28 mAb and fully stained cells within the **(A)** CD4+ and **(B)** CD8+ T-PBL gate.
(TIF)

S1 File ARRIVE guidelines checklist. (DOCX)

S2 File Statistical data. (DOC)
