# Supplementary figures and images for: Strain differences in thymic atrophy in rats immunized for EAE correlate with the clinical outcome of immunization
Source: PLoS One. 2018 Aug 7;13(8):e0201848. doi: 10.1371/journal.pone.0201848 (PMC6080797; doi:10.1371/journal.pone.0201848)

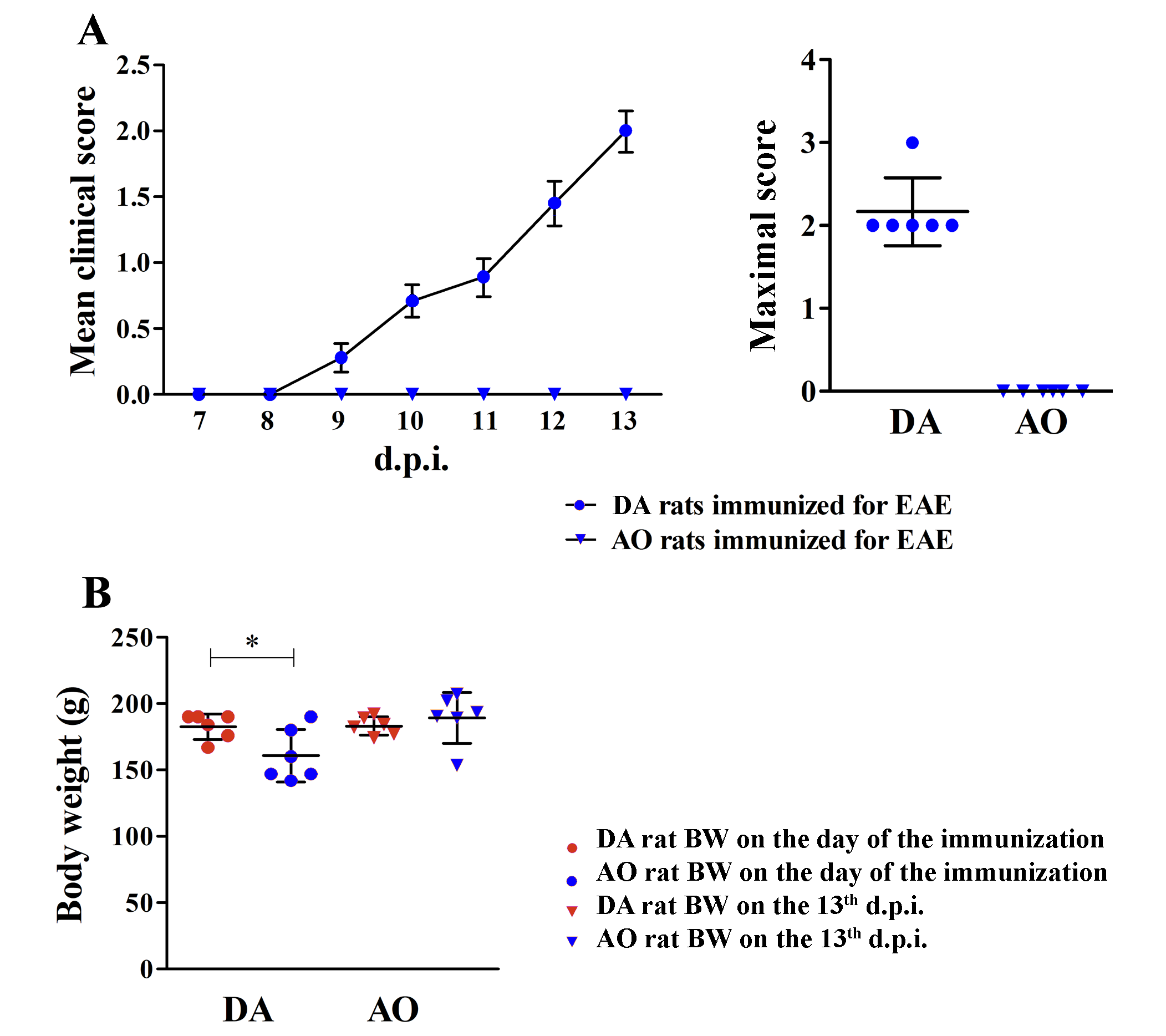

Supplement: S1 Fig — (A) Line graph indicates the mean daily clinical score of experimental autoimmune encephalomyelitis (EAE) in DA and AO rats immunized with rat spinal cord homogenate supplemented with complete Freund’s adjuvant, from the day of EAE onset to the 13th day post immunization (d.p.i.), which correponds to the peak of the disease in DA rats. Scatter plots indicate (A) the maximal neurological score of DA and AO rats immunized for EAE and (B) the body weight (BW) of DA and AO rats immunized for EAE recorded on the day of immunization and on the 13th d.p.i. Data points, means and ± SD are from one experiment of two sets of experiments with similar results (n = 6). * p<0.05. (TIF) [file pone.0201848.s001.tif]

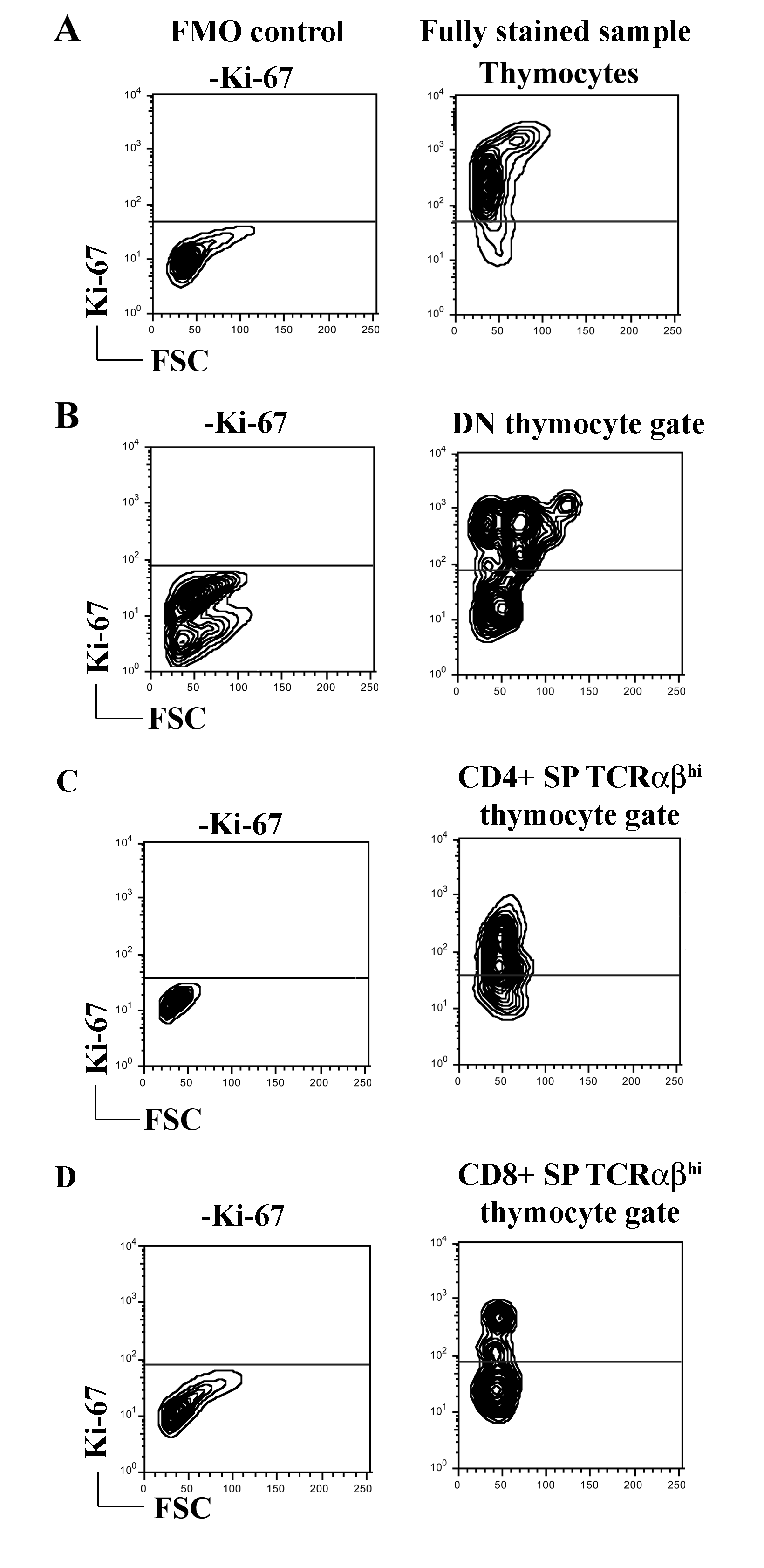

Supplement: S2 Fig — Gating strategy based on fluorescence minus one (FMO) controls for setting cut off boundaries for analysis of Ki-67 expression in Ki-67/TCRαβ/CD4/CD8 stained thymocytes from non-immunized and immunized for EAE DA and AO rats. Flow cytometry contour plots represent FMO controls without anti-Ki-67 mAb and corresponding fully stained cells within (A) thymocytes, (B) CD4-CD8- double negative (DN), (C) CD4+ single positive (SP) TCRαβhi and (D) CD8+ SP TCRαβhi thymocyte gate. (TIF) [file pone.0201848.s002.tif]

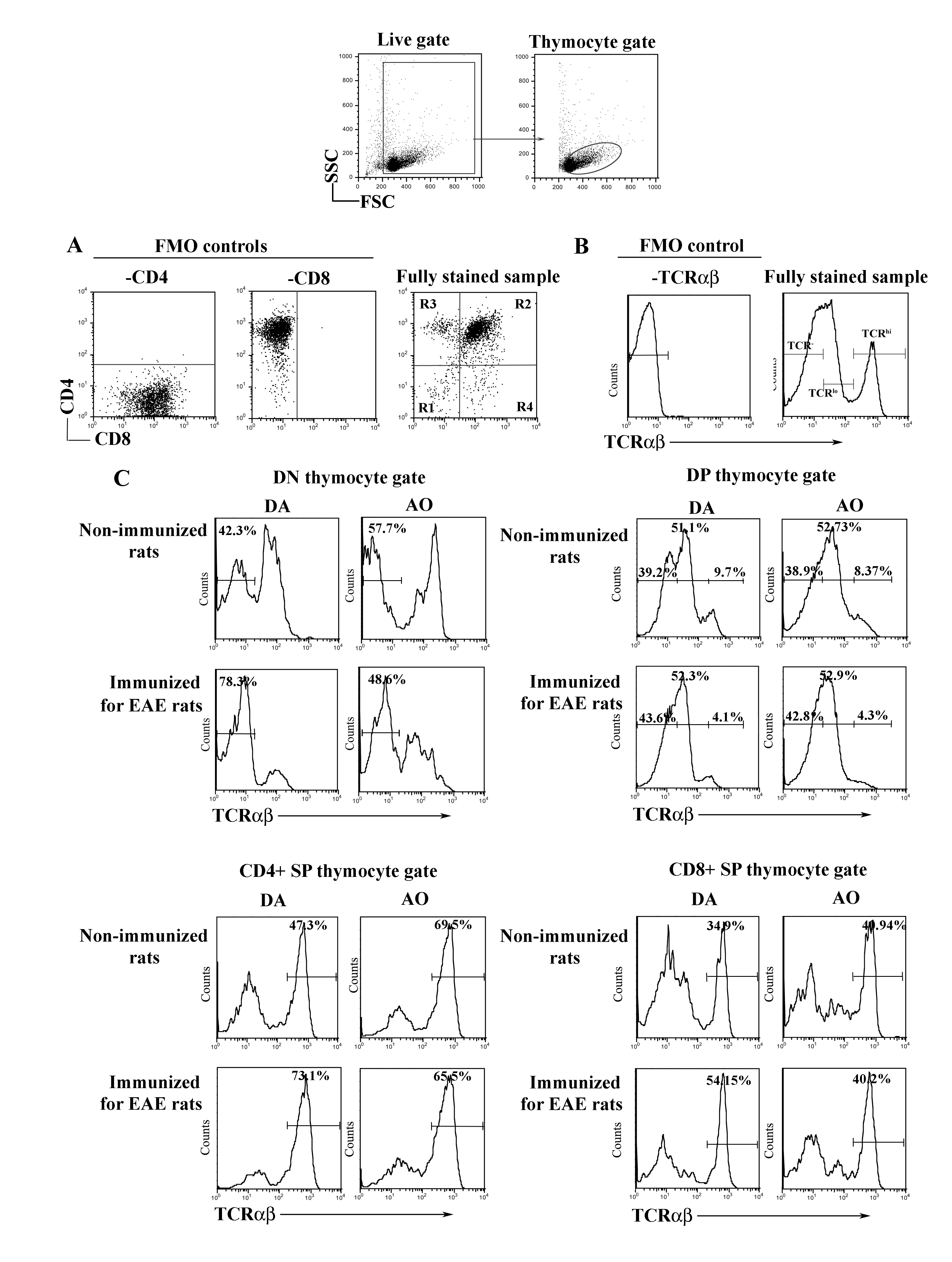

Supplement: S3 Fig — (A) Flow cytometry dot plots represent fluorescence minus one (FMO) controls without anti-CD4 or anti-CD8 mAbs and fully stained thymocytes (gated within the live gate, as shown on the appropriate flow cytometry dot plots). R1 = CD4-CD8- (double negative, DN) thymocytes; R2 = CD4+CD8+ (double positive, DP) thymocytes; R3 = CD4+ (single positive, SP) thymocytes and R4 = CD8+ SP thymocytes. (B) Flow cytometry histograms represent FMO control without anti-TCRαβ mAb and fully stained thymocytes. (C) Representative flow cytometry histograms show TCRαβ expression on DN, DP, CD4+ and CD8+ SP thymocytes (gated as shown in A) of non-immunized and immunized for EAE DA and AO rats. (TIF) [file pone.0201848.s003.tif]

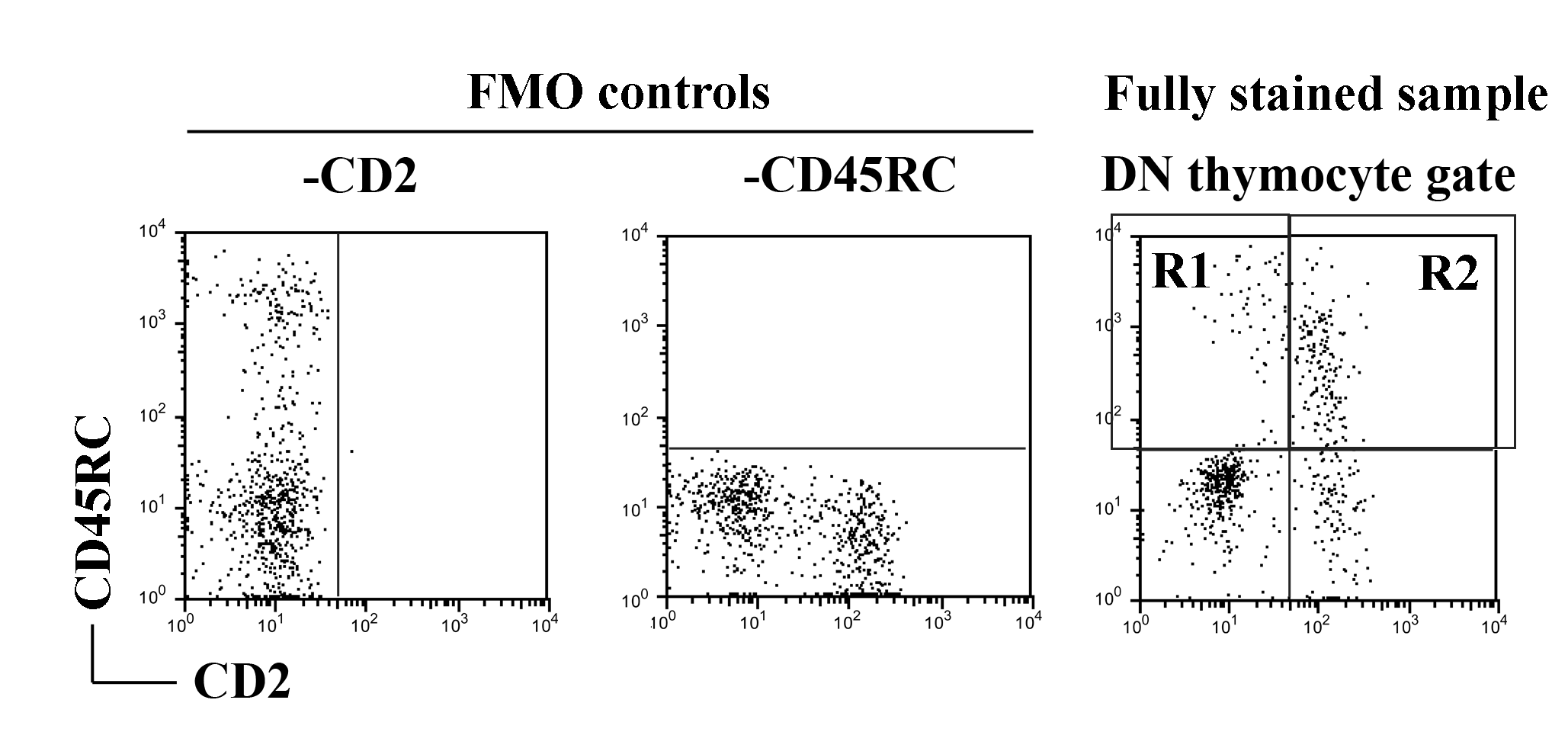

Supplement: S4 Fig — Gating strategy based on fluorescence minus one (FMO) controls for setting cut off boundaries for analysis of CD2/CD45RC expression on CD4/CD8 stained thymocytes from non-immunized and immunized for EAE DA and AO rats. Flow cytometry dot plots represent FMO controls without anti-CD2 or anti-CD45RC mAbs and fully stained cells within the CD4-CD8- double negative (DN) thymocyte gate (gated as shown in S3A Fig). R1 = CD45RC+CD2- DN thymocytes; R2 = CD45RC+CD2+ DN thymocytes. (TIF) [file pone.0201848.s004.tif]

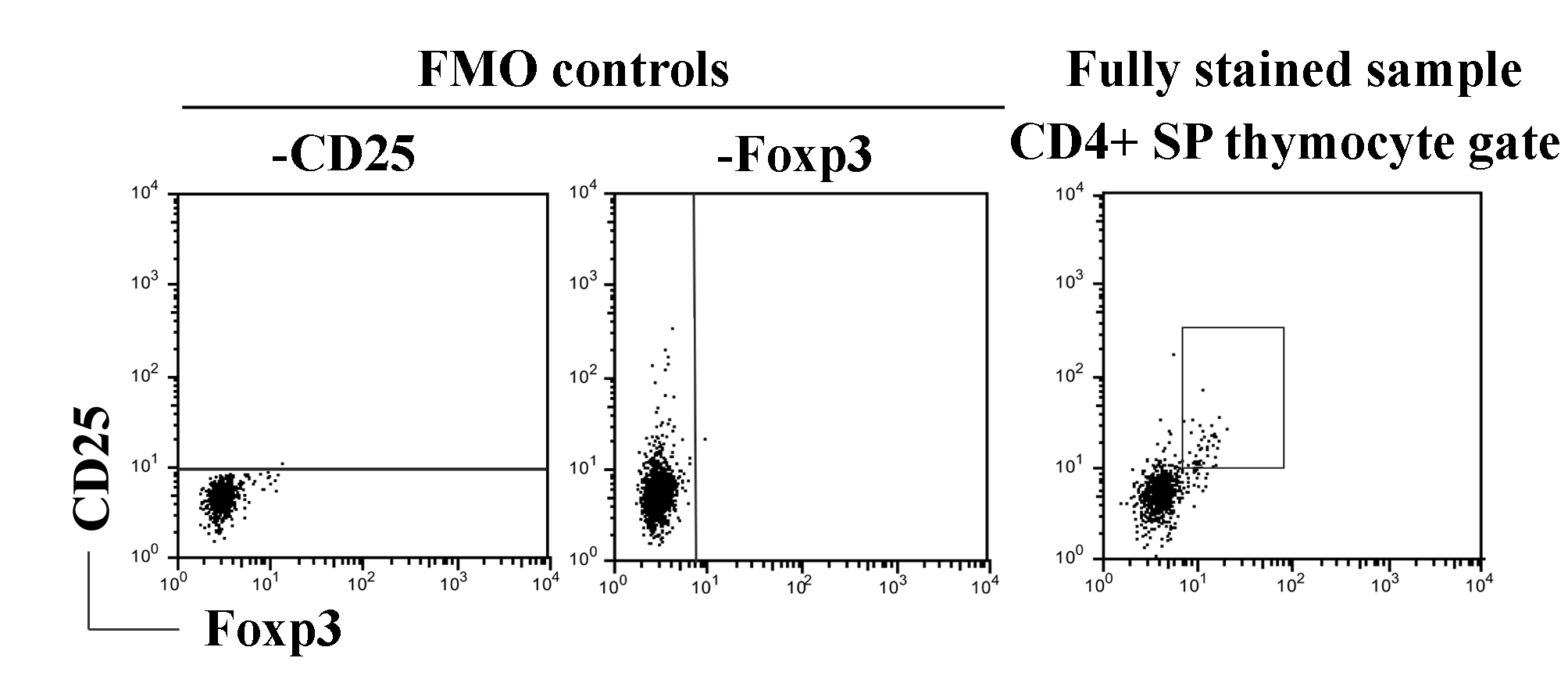

Supplement: S5 Fig — Gating strategy based on fluorescence minus one (FMO) controls for setting cut off boundaries for analysis of CD4/CD25/Foxp3 expression on thymocytes from non-immunized and immunized for EAE DA and AO rats. Flow cytometry dot plots represent FMO controls without anti-CD25 or anti-Foxp3 mAbs and fully stained cells within the CD4+ SP thymocyte gate (gated as shown in S3A Fig). (TIF) [file pone.0201848.s005.tif]

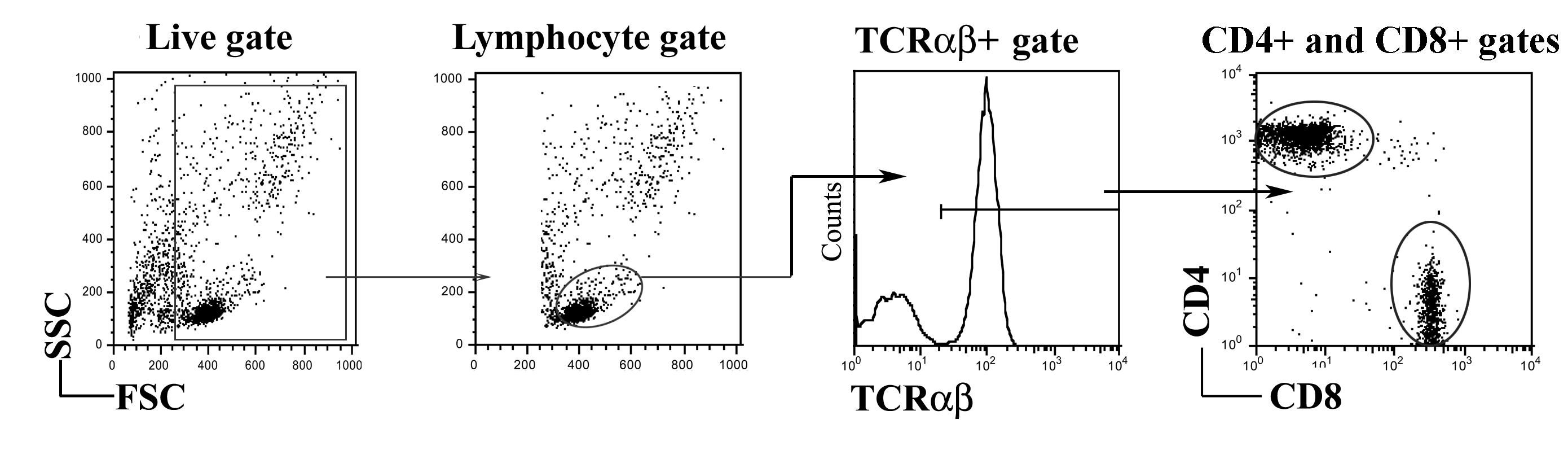

Supplement: S6 Fig — Gating strategy for analysis of TCRαβ/CD4/CD8 stained T-peripheral blood lymphocytes (T-PBLs) from non-immunized and immunized for EAE DA and AO rats. CD4 and CD8 expression was analyzed in T-PBLs (TCRαβ+ cells), gated as shown on the flow cytometry histogram. TCRαβ+ cells were gated within live lymphocytes, as shown on the appropriate flow cytometry dot plots. (TIF) [file pone.0201848.s006.tif]

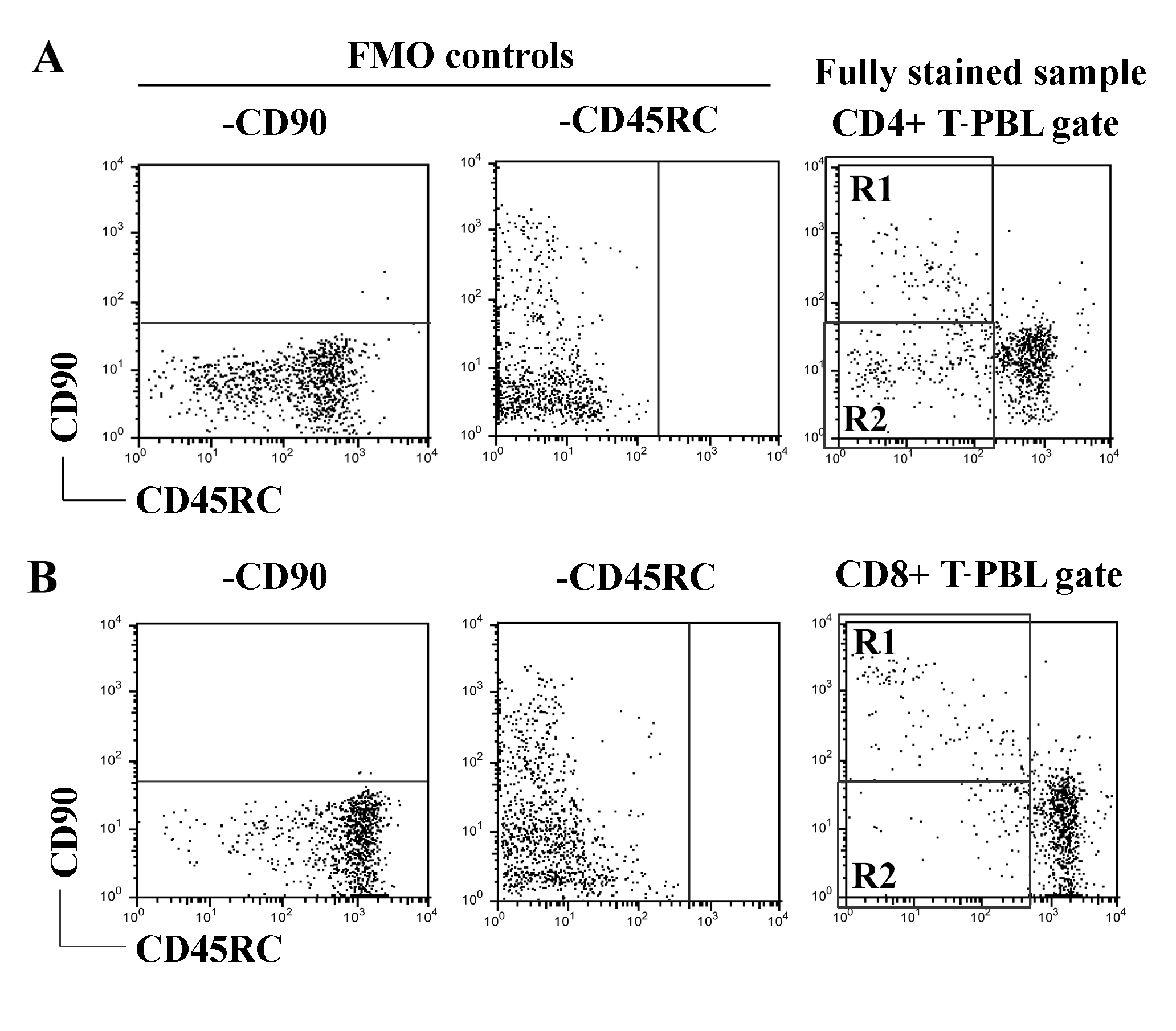

Supplement: S7 Fig — Gating strategy based on fluorescence minus one (FMO) controls for setting cut off boundaries for analysis of CD90/CD45RC expression on CD4+ and CD8+ T-peripheral blood lymphocytes (T-PBLs) from non-immunized and immunized for EAE DA and AO rats. Flow cytometry dot plots represent FMO controls without anti-CD90 or anti-CD45RC mAbs and fully stained cells within (A) CD4+ and (B) CD8+ T-PBLs (gating strategies for CD4+ and CD8+ T-PBLs are displayed in S6 Fig). R1 = CD45RC-CD90+ cells (RTEs); R2 = CD45RC-CD90- cells (memory phenotype). (TIF) [file pone.0201848.s007.tif]

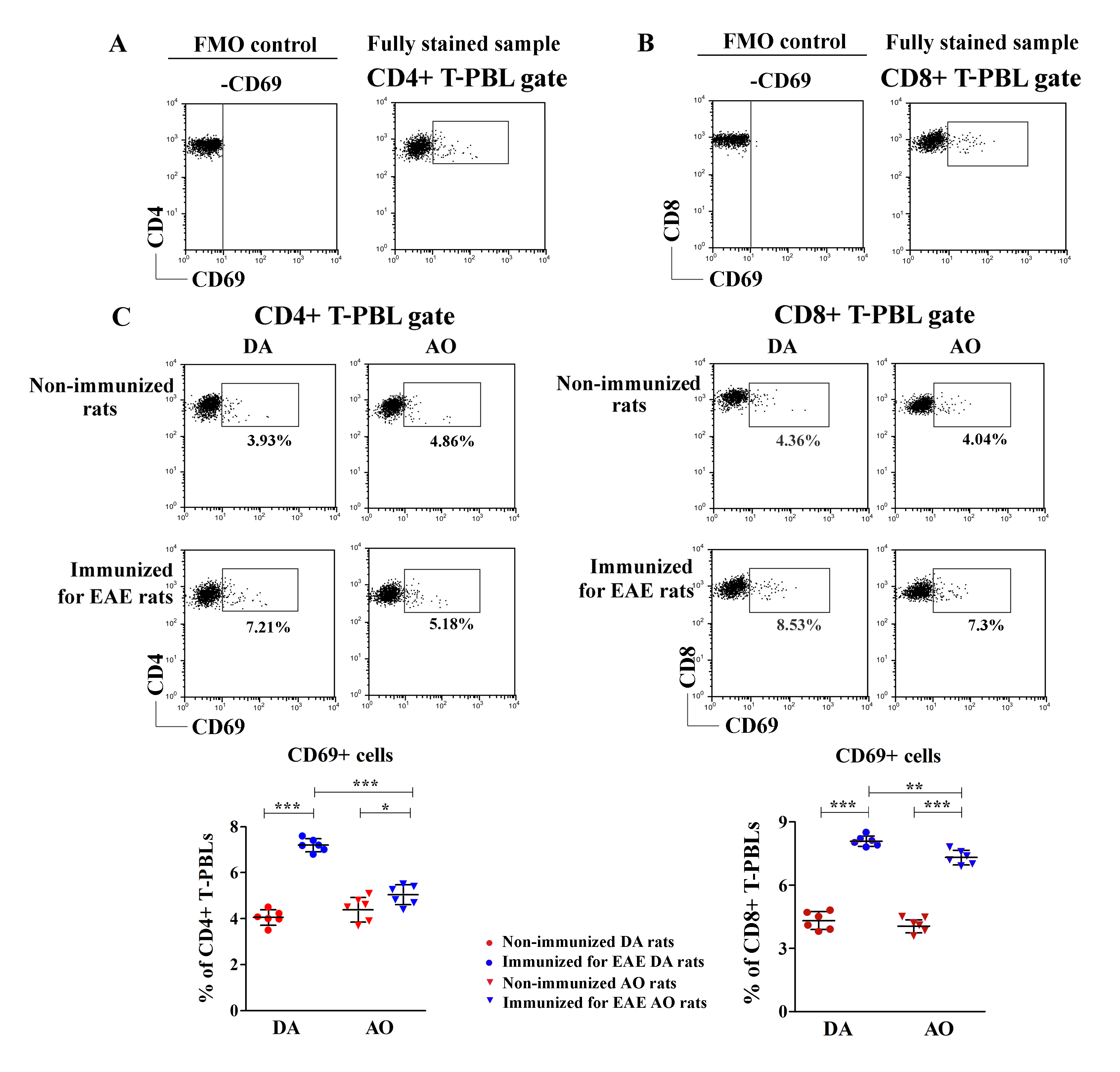

Supplement: S8 Fig — (A,B) Gating strategy based on fluorescence minus one (FMO) controls for setting cut off boundaries for analysis of CD69 expression on CD4+ and CD8+ T-peripheral blood lymphocytes (T-PBLs) from non-immunized and immunized for EAE DA and AO rats. Flow cytometry dot plots represent FMO controls without anti-CD69 Ab and fully stained cells within (A) CD4+ and (B) CD8+ T-PBLs (gating strategies for CD4+ and CD8+ T-PBLs are displayed in S6 Fig). (C) Flow cytometry dot plots show CD69 staining of CD4+ and CD8+ T-PBLs of non-immunized and immunized for EAE DA and AO rats. Scatter plots indicate the frequency of CD69+ cells within CD4+ and CD8+ T-PBLs. Two way ANOVA showed significant interaction between the effect of strain and immunization for the frequency of CD69+ cells within CD4+ T-PBLs (F(1,20) = 56.89, p<0.001). Data points, means and ± SD are from one of two experiments with similar results (n = 6). * p<0.05; ** p<0.01; *** p<0.001. (TIF) [file pone.0201848.s008.tif]

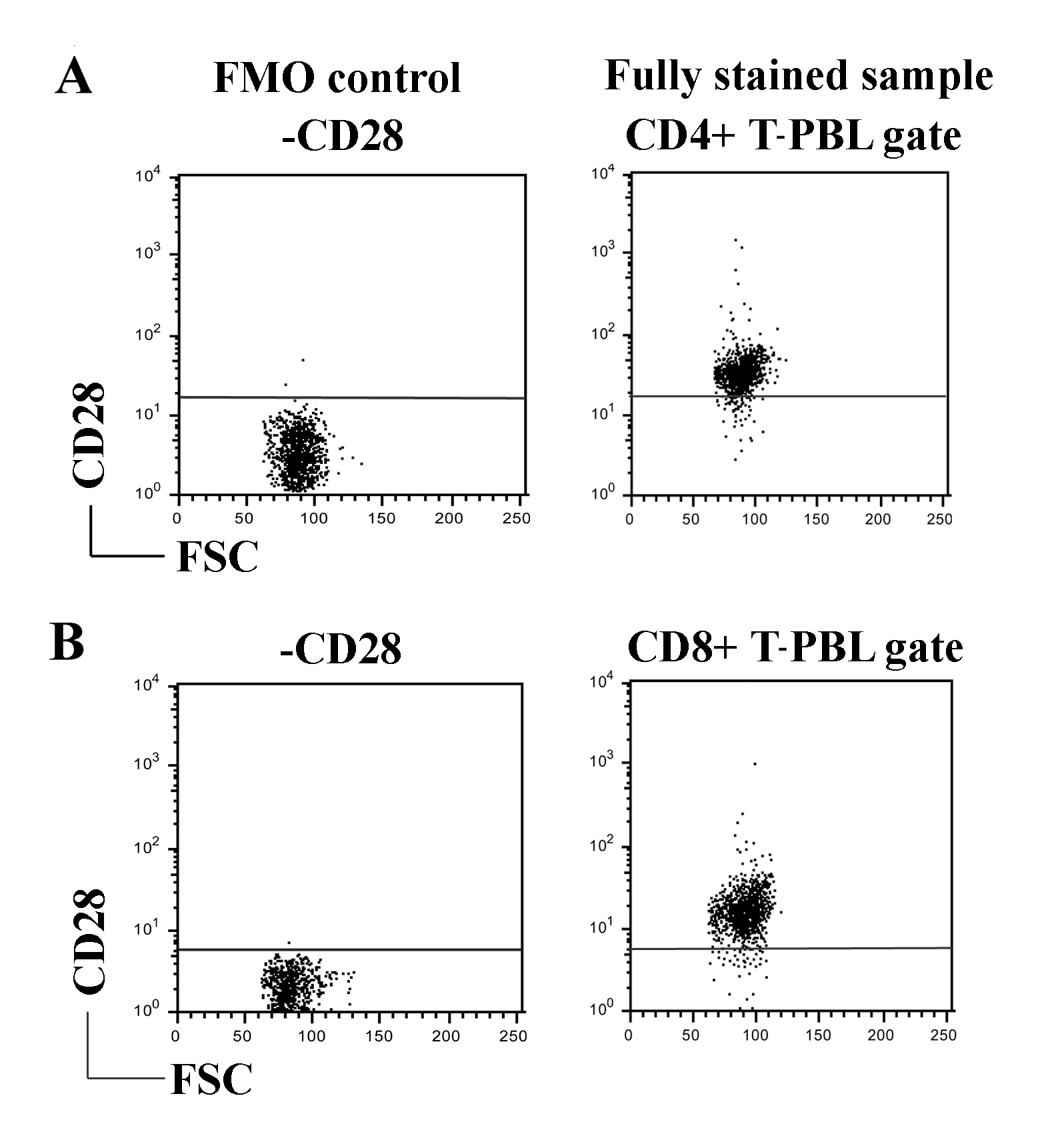

Supplement: S9 Fig — Gating strategy based on fluorescence minus one (FMO) controls for setting cut off boundaries for analysis of CD28 expression on (A) TCRαβ/CD4 and (B) TCRαβ/CD8 stained T-peripheral blood lymphocytes (T-PBLs) from non-immunized and immunized for EAE DA and AO rats (gating strategies for CD4+ and CD8+ T-PBLs are displayed in S6 Fig). Flow cytometry dot plots represent FMO controls without anti-CD28 mAb and fully stained cells within the (A) CD4+ and (B) CD8+ T-PBL gate. (TIF) [file pone.0201848.s009.tif]
