# Supplementary material for: Strain differences in thymic atrophy in rats immunized for EAE correlate with the clinical outcome of immunization
Source: PLoS One. 2018 Aug 7;13(8):e0201848. doi: 10.1371/journal.pone.0201848 (PMC6080797; doi:10.1371/journal.pone.0201848)

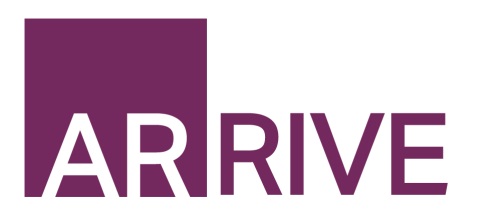


The ARRIVE Guidelines Checklist

Animal Research: Reporting In Vivo Experiments

Carol Kilkenny^1^, William J Browne^2^, Innes C Cuthill^3^, Michael Emerson^4^ and Douglas G Altman^5^

*^1^The National Centre for the Replacement, Refinement and Reduction of Animals in Research, London, UK, ^2^School of Veterinary Science, University of Bristol, Bristol, UK, ^3^School of Biological Sciences, University of Bristol, Bristol, UK, ^4^National Heart and Lung Institute, Imperial College London, UK, ^5^Centre for Statistics in Medicine, University of Oxford, Oxford, UK.*

|  | | ITEM | RECOMMENDATION | Section/ Paragraph |
| --- | --- | --- | --- | --- |
| 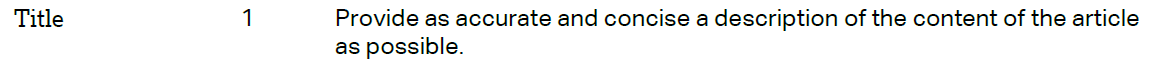 | | | Title |  |
| 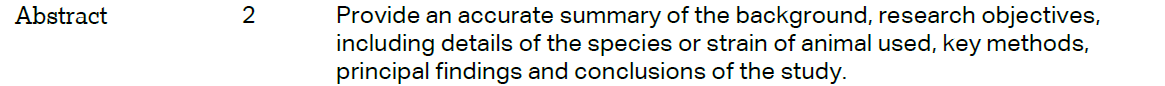 | | | Abstract |  |
| INTRODUCTION | | |  |  |
| 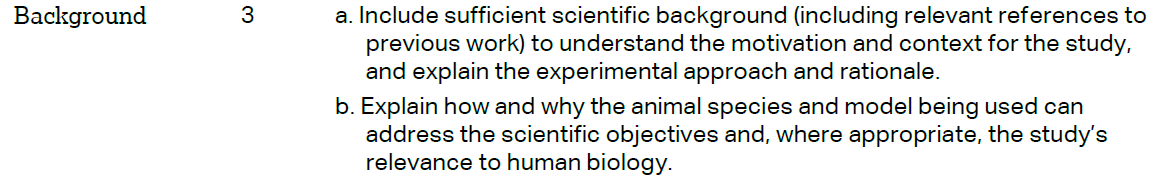 | | | Paragraph 1-8  Paragraph 4,5,8 |  |
| 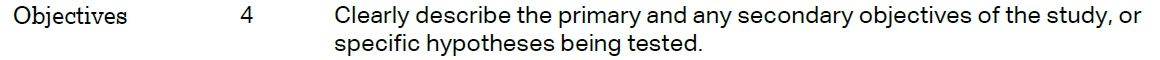 | | | Paragraph 9 |  |
| METHODS | | |  |  |
| 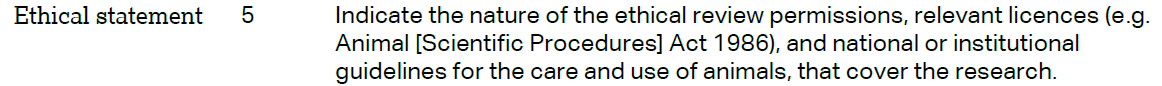 | | | Paragraph 1 |  |
| 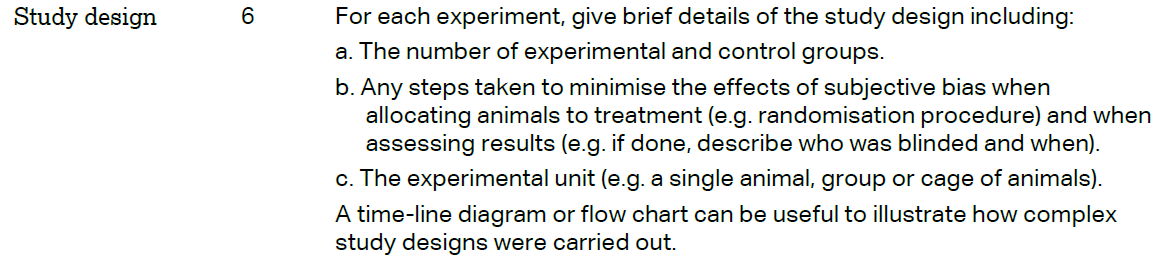 | | | Paragraph 2 |  |
| 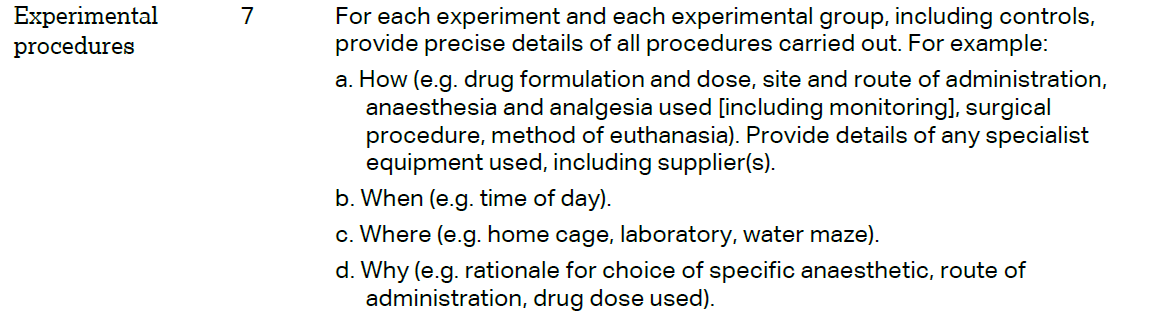 | | | Paragraph 2 |  |
| 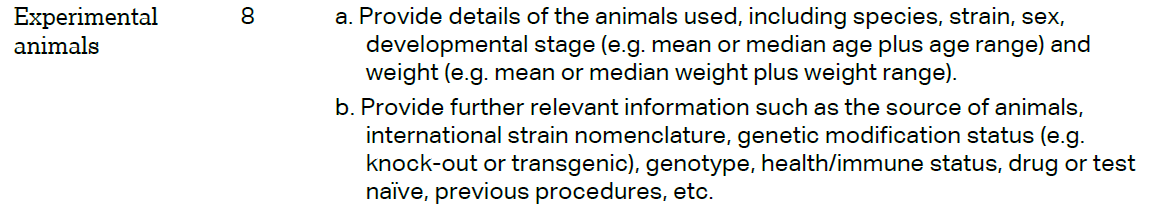 | | | Paragraph 1, S1 Fig |  |

The ARRIVE guidelines. Originally published in *PLoS Biology*, June 2010^1^

| 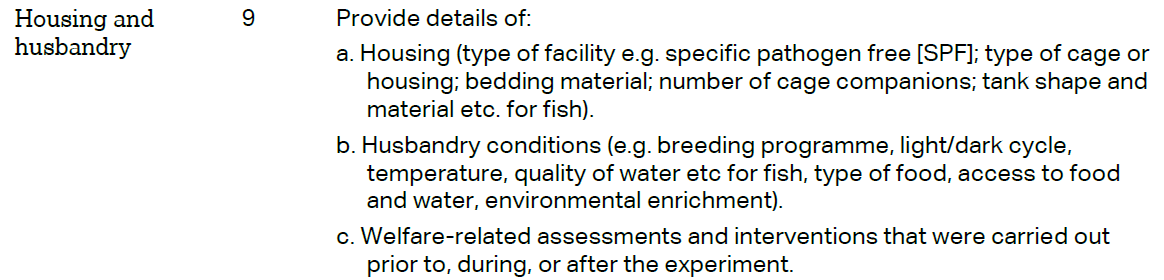 | Paragraph 1  Paragraph 1  Paragraph 2 | |
| --- | --- | --- |
| 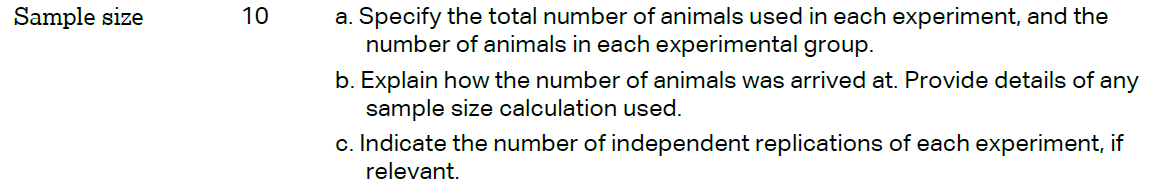 | Paragraph 2 and all figure legends | |
| 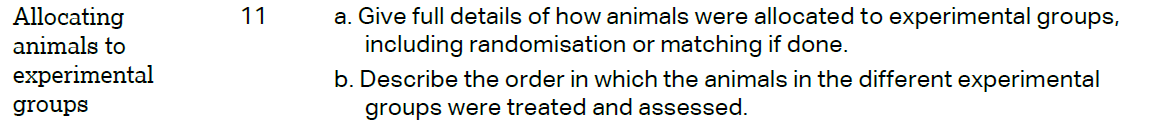 | Paragraph 2 | |
| 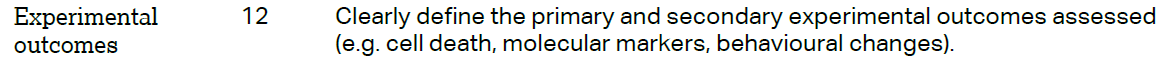 | Throughout Results | |
| 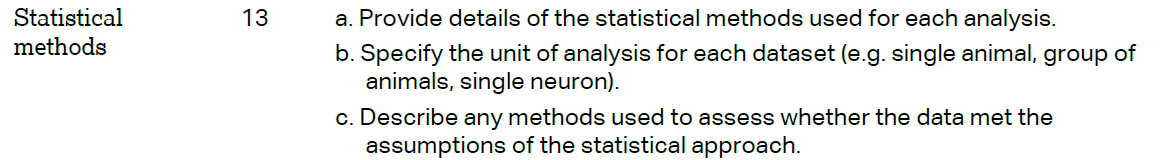 | Paragraph 11, S2 File | |
| RESULTS |  | |
| 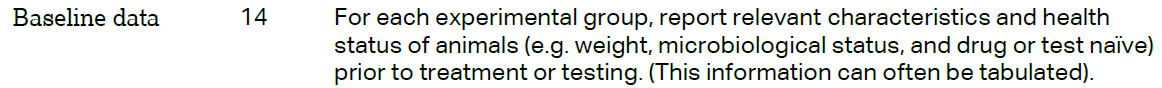 | Paragraph 1, S1 Fig | |
| 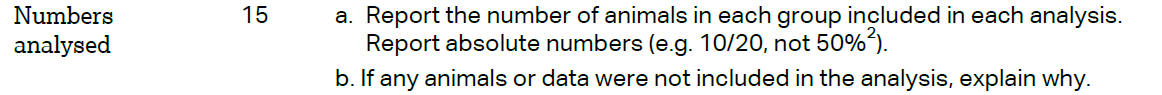 | Methods, paragraph 2 and all figure legends  No animals were excluded from the study | |
| 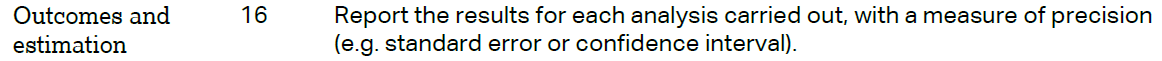 | Throughout Results, all figures and all figure legends | |
| 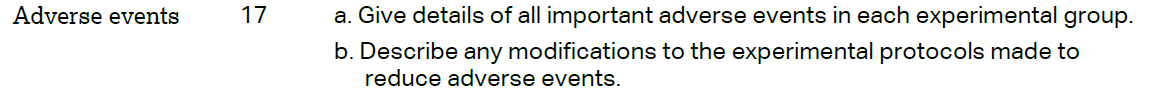 | N/A | |
| DISCUSSION |  | |
| 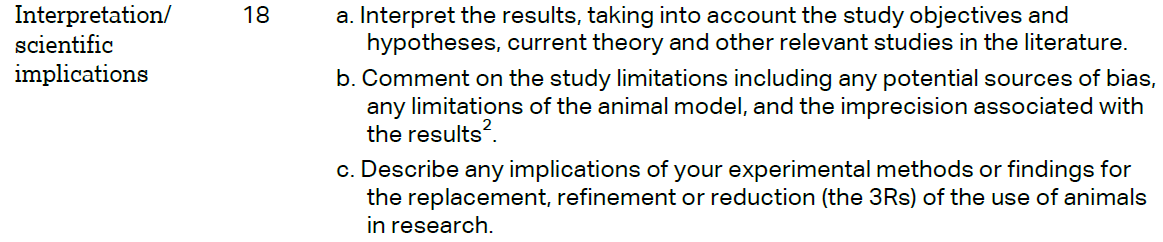 | Throughout Discussion  Introduction, paragraph 5, reference no. 26 | |
| 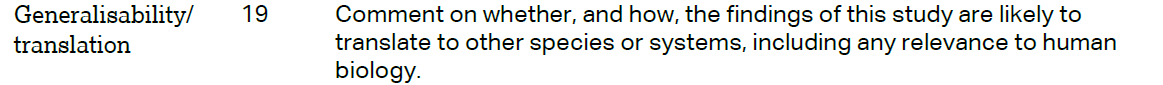 | The last paragraph | |
| 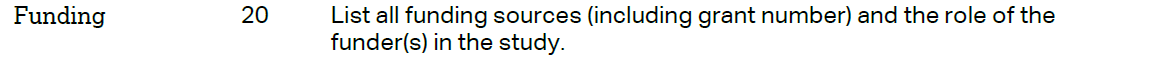 | | Declared according to PLoS One requirements |


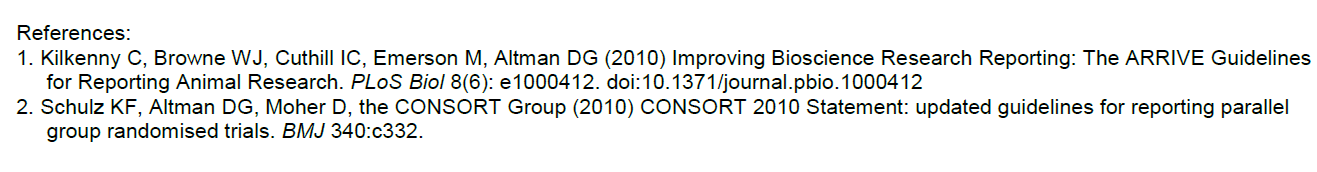

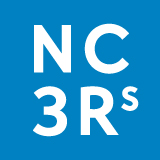

Supplement: S1 File — (DOCX) [file pone.0201848.s010.docx]
