# Supplementary material for: Strain differences in thymic atrophy in rats immunized for EAE correlate with the clinical outcome of immunization
Source: PLoS One. 2018 Aug 7;13(8):e0201848. doi: 10.1371/journal.pone.0201848 (PMC6080797; doi:10.1371/journal.pone.0201848)
Supplement: S2 File — (DOC) [file pone.0201848.s011.doc]

**Statistical analysis**

**Fig. 1. (A) Thymic weight:** Two way ANOVA (strain x immunization) showed a significant effect of strain (F_(1,20)_=20.16, p<0.001) and immunization (F_(1,20)_=26.32, p<0.001), and significant interaction between the factors (F(_1,20)_=6.954, p<0.01). **Thymocyte yield:** Two-way ANOVA (strain x immunization) revealed a significant effect of strain (F_(1,20)_=12.41, p<0.01) and immunization (F_(1,20)_=135.6, p<0.001), and a significant interaction between the factors (F_(1,20)_=69.94, p<0.001). **Normalized thymic weight:** Two-way ANOVA (strain x immunization) revealed a significant effect of strain (F_(1,20)_=22.54, p<0.001) and immunization (F_(1,20)_=26.19, p<0.001), and significant interaction between the factors (F_(1,20)_=14.63, p<0.01). **Normalized thymocyte yield:** Two-way ANOVA (strain x immunization) revealed a significant effect of strain (F_(1,20)_= 15.23, p<0.001) and immunization (F_(1,20)_=260.7, p<0.001), and significant interaction between the factors (F_(1,20)_=148.1, p<0.001). **(B) Circulating levels of IL-6:** Two-way ANOVA (strain x immunization) revealed a significant effect of strain (F_(1,20)_=71.63, p<0.001) and immunization (F_(1,20)_=63.08, p<0.001), and a significant interaction between the factors (F_(1,20)_=19.60, p<0.001). **Thymic expression of IL-6 mRNA:** Two-way ANOVA (strain x immunization) revealed a significant effect of strain (F_(1,20)_=213.1, p<0.001) and immunization (F_(1,20)_=54.64, p<0.001), and a significant interaction between the factors (F_(1,20)_=3557, p<0.001). **(C) Frequency of Ki-67+ thymocytes:** Two-way ANOVA (strain x immunization) revealed a non-significant effect of strain (F_(1,20)_=0.00005290, p>0.05) and immunization (F_(1,20)_=1.535, p>0.05), and non-significant interaction between the factors (F_(1,20)_=0.1524, p>0.05). **(D) Frequency of MC540+ thymocytes:** Two-way ANOVA (strain x immunization) revealed a non-significant effect of strain (F_(1,20)_=1.046, p>0.05), a significant effect of immunization (F_(1,20)_=92.87, p<0.001), and significant interaction between the factors (F_(1,20)_= 19.37, p<0.001). **(E)** **Thymic expression of IL-7 mRNA:** Two-way ANOVA (strain x immunization) revealed a significant effect of strain (F_(1,20)_=91.26, p<0.001) and immunization (F_(1,20)_=121.0, p<0.001), and non-significant interaction between the factors (F_(1,20)_=0.6160, p>0.05).

**Fig. 2. Frequency of CD4−CD8− (double negative, DN) thymocytes:** Two-way ANOVA (strain x immunization) showed a significant effect of strain (F_(1,20)_=4.725, p<0.05) and immunization (F_(1,20)_=68.34, p<0.001) and non-significant interaction between the factors (F_(1,20)_=0.5944, p>0.05). **Frequency of CD4+CD8+ (double positive, DP) thymocytes:** Two-way ANOVA (strain x immunization) revealed a significant effect of strain (F_(1,20)_=33.46, p<0.001) and immunization (F_(1,20)_=23.64, p<0.001), and a non-significant interaction between the factors (F_(1,20)_=1.359, p>0.05). **Frequency of CD4+ (single positive, SP) thymocytes:** Two-way ANOVA (strain x immunization) revealed a significant effect of strain (F_(1,20)_=38.37, p<0.001) and immunization (F_(1,20)_=88.07, p<0.001), and significant interaction between the factors (F_(1,20)_=9.376, p<0.001). **Frequency of CD8+ SP thymocytes:** Two-way ANOVA (strain x immunization) showed a non-significant effect of strain (F_(1,20)_=0.50, p>0.05), a significant main effect of immunization (F_(1,20)_=18.71, p<0.001), and non-significant interaction between the factors (F_(1,20)_=0.0008477, p>0.05).

**Fig. 3.** **(A) Frequency of CD4−CD8− (double negative, DN) TCRαβ^-^ thymocytes:** Two-way ANOVA (strain x immunization) showed a significant effect of strain (F_(1,20)_=43.65, p<0.001) and immunization (F_(1,20)_=229.4, p<0.001), and significant interaction between factors (F_(1,20)_=73.35, p<0.001). **(B) Frequency of** **CD45RC+CD2- DN thymocytes:** Two-way ANOVA (strain x immunization) showed a significant effect of strain (F_(1,20)_=73.48, p<0.001) and immunization (F_(1,20)_=94.89, p<0.001), and significant interaction between the factors (F_(1,20)_=63.06, p<0.001). **Frequency of** **CD45RC+CD2+ DN thymocytes:** Two-way ANOVA (strain x immunization) showed a significant effect of strain (F_(1,20)_=51.72, p<0.001) and immunization (F_(1,20)_=161.7, p<0.001), and significant interaction between the factors (F_(1,20)_=160.3, p<0.001). **(C) Expression of CXCL12 mRNA:** Two-way ANOVA (strain x immunization) showed a significant effect of strain (F_(1,20)_=23.08, p<0.001) and immunization (F_(1,20)_=23.39, p<0.001), and significant interaction between the factors (F_(1,20)_=49.48, p<0.001). **(D) Frequency of MC540+ cells within DN thymocytes:** Two-way ANOVA (strain x immunization) showed a significant effect of strain (F_(1,20)_=25.26, p<0.001) and immunization (F_(1,20)_=742.2, p<0.001), and significant interaction between the factors (F_(1,20)_=232.1, p<0.001). **(E) Frequency of Ki-67+ cells within DN thymocytes:** Two-way ANOVA (strain x immunization) showed a significant main effect of strain (F_(1,20)_=17.09, p<0.001), non-significant effect of immunization (F_(1,20)_=1.814, p>0.05) and non-significant interaction between the factors (F_(1,20)_=0.4229, p>0.05).

**Fig. 4. (A) Frequency of DP TCRαβ^-^ thymocytes:** Two-way ANOVA (strain x immunization) showed a non-significant effect of strain (F_(1,20)_=3.923, p>0.05), a significant main effect of immunization (F_(1,20)_=33.23, p<0.001), and non-significant interaction between the factors (F_(1,20)_=0.01774, p>0.05). **Frequency of DP TCRαβ^lo^ thymocytes:** Two-way ANOVA (strain x immunization) showed a significant main effect of strain (F_(1,20)_=4.483, p<0.05), and a non-significant effect of immunization (F_(1,20)_=2.029, p>0.05) and non-significant interaction between the factors (F_(1,20)_=0.1241, p>0.05). **Frequency of DP TCRαβ^hi^ thymocytes:** Two-way ANOVA (strain x immunization) showed a non-significant effect of strain (F_(1,20)_=2.881, p>0.05), a significant effect of immunization (F_(1,20)_=659.6, p<0.001) and significant interaction between the factors (F_(1,20)_=6.987, p<0.05). **(B) Fold change of CD90 MFI ratio of DP TCR^lo^ thymocytes:** Two-way ANOVA (strain x immunization) showed a significant effect of strain (F_(1,20)_=4.841, p<0.05) and immunization (F_(1,20)_=74.72, p<0.001), and non-significant interaction between the factors (F_(1,20)_=0.8645, p>0.05). **(C) Fold change of CD90 MFI ratio of DP TCR^hi^ thymocytes:** Two-way ANOVA (strain x immunization) showed a non-significant effect of strain (F_(1,20)_=0.03175, p>0.05), a significant main effect of immunization (F_(1,20)_=73.14, p<0.001), and non-significant interaction between the factors (F_(1,20)_=2.032, p>0.05).

**Fig. 5** **(A) Frequency of CD4+ SP TCRαβ^hi^ thymocytes:** Two-way ANOVA (strain x immunization) showed a significant effect of strain (F_(1,20)_=14.87, p<0.01) and immunization (F_(1,20)_=84.49, p<0.001), and significant interaction between the factors (F_(1,20)_=55.12, p<0.001). **Frequency of CD8+ SP TCRαβ^hi^ thymocytes:** Two-way ANOVA (strain x immunization) showed a significant effect of strain (F_(1,20)_=4.756, p<0.05), a non-significant effect of immunization (F_(1,20)_=2.633, p>0.05), and significant interaction between the factors (F_(1,20)_=20.97, p<0.001). **(B)** **Frequency of Ki-67+ cells within CD4+ SP TCR^hi^ thymocytes:** Two-way ANOVA (strain x immunization) showed a significant effect of strain (F_(1,20)_=34.80, p<0.001) and immunization (F_(1,20)_=28.50, p<0.001), and a non-significant interaction between the factors (F_(1,20)_=0.009996, p>0.05). **Frequency of Ki-67+ cells within CD8+ SP TCR^hi^ thymocytes:** Two-way ANOVA (strain x immunization) showed a significant effect of strain (F_(1,20)_=21.39, p<0.001), and immunization (F_(1,20)_=32.81, p<0.001) and a non-significant interaction between the factors (F_(1,20)_=0.00114, p>0.05). **(C) Number of CD4+ SP TCR^hi^ thymocytes:** Two-way ANOVA (strain x immunization) showed a significant effect of strain (F_(1,20)_=203.9, p<0.001) and immunization (F_(1,20)_=359.5, p<0.001), and significant interaction between the factors (F_(1,20)_=229.8, p<0.001). **Number of CD8+ SP TCR^hi^ thymocytes:** Two-way ANOVA (strain x immunization) showed a significant effect of strain (F_(1,20)_=23.38, p<0.001) and immunization (F_(1,20)_=231.0, p<0.001), and significant interaction between the factors (F_(1,20)_=64.32, p<0.001).

**Fig. 6. (A) Frequency of CD4+CD25+Foxp3+ thymocytes:** Two way ANOVA (strain x immunization) showed a significant effect of strain (F_(1,20)_=2742, p<0.001) and immunization (F_(1,20)_=14840, p<0.001), and significant interaction between the factors (F_(1,20)_=152.2, p<0.001). **Number of CD4+CD25+Foxp3+ thymocytes:** Two way ANOVA (strain x immunization) showed a significant effect of strain (F_(1,20)_=21.90, p<0.001) and immunization (F_(1,20)_=314.4, p<0.001), and significant interaction between the factors (F_(1,20)_=7.296, p<0.05). **(B) IL-2 mRNA expression in thymic tissue:** Two way ANOVA (strain x immunization) showed a non-significant effect of strain (F_(1,20)_=1.212, p>0.05), a significant effect of immunization (F_(1,20)_=84.15, p<0.001) and significant interaction between the factors (F_(1,20)_=28.31, p<0.001). **IL-15 mRNA expression in thymic tissue:** Two way ANOVA (strain x immunization) showed a significant effect of strain (F_(1,20)_=16.00, p<0.001) and immunization (F_(1,20)_=177.8, p<0.001), and significant interaction between the factors (F_(1,20)_=64.0, p<0.001).

**Fig. 7. (A)** **Number of T-peripheral blood lymphocytes (T-PBLs):** Two way ANOVA (strain x immunization) showed a significant effect of strain (F_(1,20)_=6.257, p<0.05) and immunization (F_(1,20)_=39.37, p<0.001), and significant interaction between the factors (F_(1,20)_=26.16, p<0.001). **(B) Frequency of CD4+ T-PBLs:** Two way ANOVA (strain x immunization) showed a non-significant effect of strain (F_(1,20)_=1.931, p>0.05), a significant main effect of immunization (F_(1,20)_=15.97, p<0.001) and non-significant interaction between the factors (F_(1,20)_=2.062, p>0.05). **Frequency of CD8+ T-PBLs:** Two way ANOVA (strain x immunization) showed a significant effect of strain (F_(1,20)_=119.7, p<0.001), a non-significant effect of immunization (F_(1,20)_=1.043, p>0.05) and significant interaction between the factors (F_(1,20)_=29.50, p<0.001). **Number of CD4+ T-PBLs:** Two way ANOVA (strain x immunization) showed a non-significant effect of strain (F_(1,20)_=3.540, p>0.05), a significant effect of immunization (F_(1,20)_=32.29, p<0.001) and significant interaction between the factors (F_(1,20)_=69.30, p<0.001). **Number of CD8+ T-PBLs:** Two way ANOVA (strain x immunization) showed a significant effect of strain (F_(1,20)_=135.2 p<0.001) and immunization (F_(1,20)_=6.301, p<0.05), and significant interaction between the factors (F_(1,20)_=193.9, p<0.001).

**Fig. 8. (A) Frequency of CD90+CD45RC- cells within CD4+ T-peripheral blood lymphocytes (T-PBLs):** Two way ANOVA (strain x immunization) showed a significant effect of strain (F_(1,20)_=35.29, p<0.001) and immunization (F_(1,20)_=252.6, p<0.001), and non-significant interaction between the factors (F_(1,20)_=0.02703, p>0.05). **Frequency of CD90-CD45RC- cells within CD4+ T-PBLs:** Two way ANOVA (strain x immunization) showed a significant effect of strain (F_(1,20)_=39.58, p<0.001) and immunization (F_(1,20)_=67.11, p<0.001), and significant interaction between the factors (F_(1,20)_=28.52, p<0.001). **Frequency of CD90+CD45RC- cells within CD8+ T-PBLs:** Two-way ANOVA (strain x immunization) revealed a significant effect of strain (F_(1,20)_=19.15, p<0.001) and immunization (F_(1,20)_=81.37, p<0.001), and non-significant interaction between the factors (F_(1,20)_=0.9072, p>0.05). **Frequency of CD90-CD45RC- cells within CD8+ T-PBLs:** Two way ANOVA (strain x immunization) showed a significant effect of strain (F_(1,20)_=68.17, p<0.001) and immunization (F_(1,20)_=53.69, p<0.001), and non-significant interaction between the factors (F_(1,20)_=0.4731, p>0.05).

**Fig. 9. (A) Frequency of CD25+ cells within CD4+ T-peripheral blood lymphocytes (T-PBLs):** Two way ANOVA (strain x immunization) showed a significant effect of strain (F_(1,20)_=17.94, p<0.001) and immunization (F_(1,20)_=67.20, p<0.001), and significant interaction between the factors (F_(1,20)_= 22.73, p<0.001). **Frequency of CD25+ cells within CD8+ T-PBLs:** Two way ANOVA (strain x immunization) showed a non-significant effect of strain (F_(1,20)_=0.5407, p>0.05), a significant main effect of immunization (F_(1,20)_=280.6, p<0.001), and non-significant interaction between the factors (F_(1,20)_=2.238, p>0.05). **Frequency of Ki-67+ cells within CD4+CD28+ T-PBLs:** Two way ANOVA (strain x immunization) showed a significant effect of strain (F_(1,20)_=47.50, p<0.001) and immunization (F_(1,20)_=28.83, p<0.001), and significant interaction between the factors (F_(1,20)_=30.04, p<0.001). **Ffrequency of Ki-67+ cells within CD8+CD28+ T-PBLs:** Two way ANOVA (strain x immunization) showed a non-significant effect of strain (F_(1,20)_=1.761, p>0.05), a significant effect of immunization (F_(1,20)_=142.2, p<0.001), and significant interaction between the factors (F_(1,20)_=7.182, p<0.05).

**Fig. 10. (A) Frequency of CD28- cells within CD4+ T-peripheral blood lymphocytes (T-PBLs):** Two way ANOVA (strain x immunization) showed a significant effect of strain (F_(1,20)_=39.68, p<0.001) and immunization (F_(1,20)_=213.1, p<0.001), and significant interaction between the factors (F_(1,20)_=125.8, p<0.001). **Frequency of CD28- cells within CD8+ T-PBLs:** Two way ANOVA (strain x immunization) showed a significant effect of strain (F_(1,20)_=64.72, p<0.001) and immunization (F_(1,20)_=208.2, p<0.001), and significant interaction between the factors (F_(1,20)_=44.12, p<0.001). **(B) Fold change of p16^INK4a^ MFI ratio of CD4+ T-PBLs:** Two way ANOVA (strain x immunization) showed a significant effect of strain (F_(1,20)_=25.73, p<0.001) and immunization (F_(1,20)_=14.95, p<0.001) and non-significant interaction between the factors (F_(1,20)_=4.208, p>0.05). **Fold change of p16^INK4a^ MFI ratio of CD8+ T-PBLs:** Two-way ANOVA (strain x immunization) showed a significant effect of strain (F_(1,20)_=18.01, p<0.001) and immunization (F_(1,20)_=65.08, p<0.001), and non-significant interaction between the factors (F_(1,20)_=2.638, p>0.05). **(C) Circulating levels of TNF-α:** Two-way ANOVA (strain x immunization) revealed a non-significant effect of strain (F_(1,20)_=0.03518, p>0.05), significant main effect of immunization (F_(1,20)_=6.154, p<0.05), and non-significant interaction between the factors (F_(1,20)_=1.055, p>0.05).

**Fig. 11. (C) Frequency of granzyme B+ CD4+ T cells in SC mononuclear infiltrate.** Unpaired *t*-test conducted to compare the frequency of granzyme B+ CD4+ T cells in SC mononuclear infiltrate from DA (M=0.3775 ± SD=0.02469) and AO (M=0.1348 ± SD=0.01249) rats immunized for EAE revealed significant differences between the two strains (t=8.770, df=10, p<0.0001).

**S1 Fig.** **(B)** **Body weight (BW) change of DA and AO rats immunized for EAE.** Two-way ANOVA (strain x immunization) showed a significant main effect of strain (F_(1,20)_=4.903, p<0.05), non-significant effect of immunization (F_(1,20)_=1.458, p>0.05) and non-significant interaction between the factors (F_(1,20)_=4.164, p>0.05).

**S8 Fig. (C) Frequency of CD69+ cells within CD4+ T-peripheral blood lymphocytes (T-PBLs):** Two-way ANOVA (strain x immunization) showed a significant effect of strain (F_(1,20)_=30.28, p<0.001) and immunization (F_(1,20)_=133.0, p<0.001), and significant interaction between the factors (F_(1,20)_=56.89, p<0.001). **Frequency of CD69+ cells within CD8+ T-peripheral blood lymphocytes (T-PBLs):** Two-way ANOVA (strain x immunization) showed a significant effect of strain (F_(1,20)_=14.79, p<0.01) and immunization (F_(1,20)_=664.4, p<0.001), and non-significant interaction between the factors (F_(1,20)_=3.490, p>0.05).
